# Bacterial extracellular vesicles: biotechnological perspective for enhanced productivity

**DOI:** 10.1007/s11274-024-03963-7

**Published:** 2024-04-20

**Authors:** Laura M. Muñoz-Echeverri, Santiago Benavides-López, Otto Geiger, Mauricio A. Trujillo-Roldán, Norma A. Valdez-Cruz

**Affiliations:** 1 Departamento de Biología Molecular y Biotecnología, Instituto de Investigaciones Biomédicas, Universidad Nacional Autónoma de México AP. 70228, Ciudad de México, C.P. 04510 México; 2 Posgrado en Ciencias Biológicas, Universidad Nacional Autónoma de México, Unidad de Posgrado, Edificio D, 1° Piso, Circuito de Posgrados, Ciudad Universitaria, Coyoacán CDMX C.P. 04510 México; 3 Posgrado en Ciencias Biomédicas, Universidad Nacional Autónoma de México, Unidad de Posgrado, Edificio B, 1° Piso, Circuito de Posgrados, Ciudad Universitaria, Coyoacán CDMX C.P. 04510 México; 4 Centro de Ciencias Genómicas, Universidad Nacional Autónoma de México, Av. Universidad s/n, Cuernavaca, Morelos CP 62210 México; 5 Centro de Nanociencias y Nanotecnología, Universidad Nacional Autónoma de México, Km 107 Carretera, Tijuana-Ensenada, Baja California 22860 México

**Keywords:** Bacterial extracellular vesicles (BEVs), Vesiculation cellular response to stress, Artificial bacterial vesicles, Hypervesiculation strains

## Abstract

Bacterial extracellular vesicles (BEVs) are non-replicative nanostructures released by Gram-negative and Gram-positive bacteria as a survival mechanism and inter- and intraspecific communication mechanism. Due to BEVs physical, biochemical, and biofunctional characteristics, there is interest in producing and using them in developing new therapeutics, vaccines, or delivery systems. However, BEV release is typically low, limiting their application. Here, we provide a biotechnological perspective to enhance BEV production, highlighting current strategies. The strategies include the production of hypervesiculating strains through gene modification, bacteria culture under stress conditions, and artificial vesicles production. We discussed the effect of these production strategies on BEVs types, morphology, composition, and activity. Furthermore, we summarized general aspects of BEV biogenesis, functional capabilities, and applications, framing their current importance and the need to produce them in abundance. This review will expand the knowledge about the range of strategies associated with BEV bioprocesses to increase their productivity and extend their application possibilities.

**Graphical abstract:**

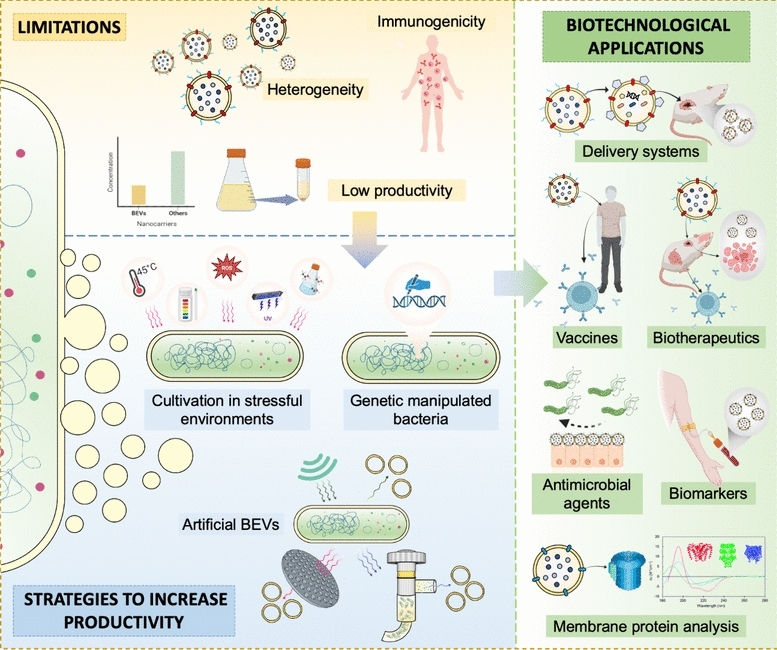

**Supplementary Information:**

The online version contains supplementary material available at 10.1007/s11274-024-03963-7.

## Introduction

Extracellular vesicles (EVs) are non-replicative membranous nanostructures released into the extracellular environment by eukaryotic cells, archaea, and bacteria (Bitto and Kaparakis-Liaskos 2017; Gill et al. 2019; Joffe et al. 2016). Particularly, Gram-negative, and Gram-positive bacteria produce extracellular vesicles (BEVs) during all phases of growth and in various environments (Bitto and Kaparakis-Liaskos 2017; Kim et al. 2015a; Klimentova et al. 2019; Koning et al. 2013; Schwechheimer and Kuehn 2015; Wang et al. 2021a, b). The release of BEVs is a conserved process recognized as a “type zero secretion system” (Bitto and Kaparakis-Liaskos 2017; Guerrero-Mandujano et al. 2017; Mozaheb and Mingeot-Leclercq 2020).

Most BEVs are spherical particles with heterogeneous sizes ranging from 10 to 400 nm in diameter (Bitto and Kaparakis-Liaskos 2017; Toyofuku et al. 2019; Uddin et al. 2020). Due to the differences in the cell wall between Gram-negative and Gram-positive bacteria, the biogenesis and composition of BEVs are different (Brown et al. 2015; Kim et al. 2015a; Toyofuku et al. 2019; Yáñez-Mó et al. 2015). Gram-negative bacteria usually release BEVs from the outer membrane (OM), called OMVs (Outer Membrane Vesicles) (Hu et al. 2020; Schwechheimer and Kuehn 2015), although the formation of outer-inner membrane vesicles (O-IMVs) has also been described (Gill et al. 2019; Pérez-Cruz et al. 2015; Toyofuku et al. 2019). OMVs and O-IMVs are composed of lipopolysaccharides (LPS), membrane lipids, peptidoglycan (PG), outer membrane proteins (OMPs), periplasmic proteins, and metabolites (Gill et al. 2019; Jan 2017; Pérez-Cruz et al. 2015; Thoma et al. 2018; Uddin et al. 2020). O-IMVs are additionally enriched for cytoplasmic (including nucleic acids) and inner membrane (IM) components when compared to OMVs (Gill et al. 2019). In contrast, Gram-positive bacteria release vesicles from the cell membrane (CM), called membrane vesicles (MV) (Cao and Lin 2021; Toyofuku et al. 2019), carrying as cargo nucleic acids, membrane and cytoplasmic proteins, membrane lipids, lipoteichoic acids (LTA), and other metabolites (Brown et al. 2015; Cao and Lin 2021).

BEVs play essential roles in bacterial survival, cell communication, infection, or cell–cell interaction (Caruana and Walper 2020; Liu et al. 2018; Schwechheimer et al. 2015; Zlatkov et al. 2021). Bacteria use BEVs to send information via effector molecules to target cells (Bitto and Kaparakis-Liaskos 2017; Gill et al. 2019; Yáñez-Mó et al. 2015), and depending on the recipient cell, cargo molecules can be delivered through membrane fusion or by endocytosis (Bitto et al. 2017; Cañas et al. 2016; Ñahui et al. 2021; Wolf et al. 2012; Yáñez-Mó et al. 2015). Furthermore, the delivery to specific cells could be directed by molecules located on the external surface of BEVs (Kaparakis-Liaskos and Ferrero 2015; Yáñez-Mó et al. 2015). While the lipid bilayer protects the cargo from adverse environmental conditions or degradative enzymes, allowing the transport of information (Bonnington and Kuehn 2014; Caruana and Walper 2020; Gill et al. 2019; Guerrero-Mandujano et al. 2017; Peng et al. 2020).

Due to BEV composition and ability to transport different cargo and information, their use in developing therapeutics, vaccines, and drug delivery systems has become a relevant research topic (Huang et al. 2022a; Liu et al. 2022b,c,d; Thomas et al. 2022). However, different limitations have been identified in the commercial use of BEVs, highlighting among them the low production yields (García-Manrique et al. 2018; Hahm et al. 2021; Hu et al. 2020; Morishita et al. 2021).

Different reviews explain key aspects of the biology of BEVs, as well as some research on genetic engineering, physical, chemical, and biotechnological strategies to bioengineer BEVs and/or increase their production (Brown et al. 2015; García-Manrique et al. 2018; Gnopo et al. 2017; Liu et al. 2022b,d; Richter et al. 2021a; Schwechheimer et al. 2015; Toyofuku et al. 2019). This review compiles relevant biological aspects of BEVs, covering information on biogenesis mechanisms, known biofunctions, and recent applications in developing new biotherapeutics. We discuss in detail strategies to produce and increase the release of BEVs and the effects of these strategies on the morphology, composition, and activity of the resulting structures to improve knowledge for its feasible biotechnological application, considering the need for its production in large quantities. The production strategies were divided into three categories: molecular modifications of strains, cultivation under stress conditions, and production and recovery of artificial BEVs. Therefore, the understanding of BEVs and integration of available strategies for bioprocess development focused on the abundant production of BEVs, will improve their productivity and biotechnological application.

## Biogenesis mechanisms of BEVs

**Fig. 1:**
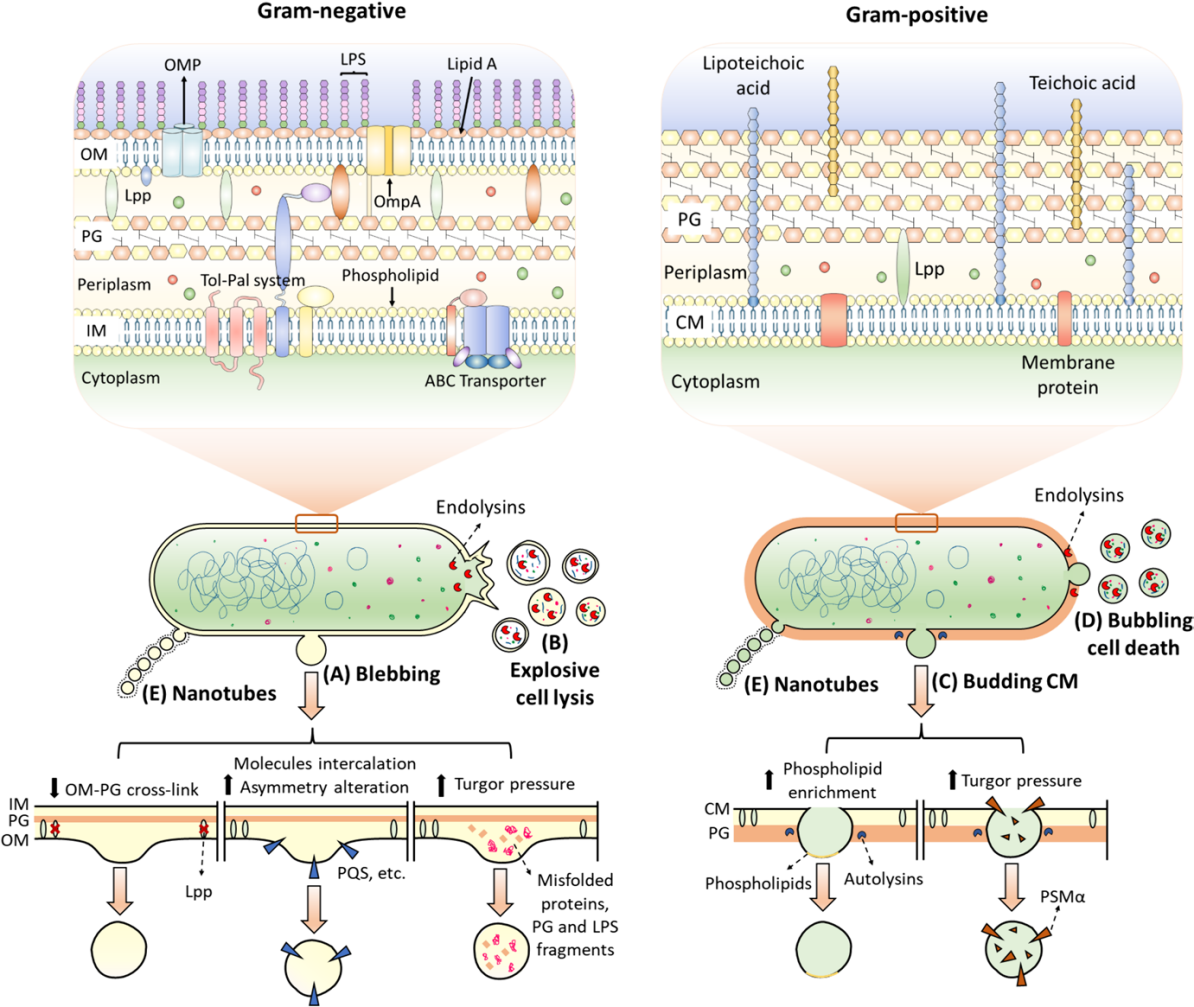
Biogenesis mechanisms of Bacterial Extracellular Vesicles (BEVs) models: (A) Blebbing and B) explosive cell lysis in Gram-negative bacteria, (C) Budding of the cellular membrane (CM) and (D) bubbling cell death in Gram-positive bacteria, and (E) nanotube formation in both. Blebbing and budding can be caused by factors such as loss of cross-links between peptidoglycan (PG) and outer membrane (OM), changes in the membrane composition, molecules intercalation like with *Pseudomonas* Quinolone Signals (PQS), or turgor pressure due to molecule accumulation in the cell wall, i.e., LPS and PG fragments, proteins, or Phenol-soluble modulins (PSMs). The explosive cell lysis and bubbling cell death are attributed to endolysins. Endolysins degrade the PG, damaging cells. Nanotubes are generated by the extrusion of the cellular membrane (CM) in Gram-positive bacteria and OM in Gram-negative bacteria. Created with Servier Medical Art resources

The biogenesis of BEVs depends on the composition of the cell wall or type of bacteria. Therefore, BEV formation mechanisms between Gram-negative and Gram-positive bacteria differ from each other, the formation of the latter being the least characterized (Briaud and Carroll 2020; Nagakubo et al. 2020; Ñahui et al. 2021). Currently, multiple mechanisms are reported to explain the generation of BEVs (Schwechheimer et al. 2013, 2014; Pathirana and Kaparakis-Liaskos 2016) divided at least into three models, not mutually exclusive: blebbing or budding of CM (Fig. 1A,C)**,** explosive cell lysis or bubbling cell death (Fig. 1B, D**),** and formation of nanotubes (Fig. 1E) (Jeong et al. 2022; Gill et al. 2019; Toyofuku et al. 2019).

### Blebbing and budding of cell membranes

In Gram-negative bacteria, blebbing occurs when the OM dissociates from the PG, protrudes to the outside, closes on itself, and detaches from the cell envelope (Schwechheimer and Kuehn 2015; Toyofuku et al. 2019). Blebbing is the main model of OMVs formation (Nagakubo et al. 2020; Toyofuku et al. 2019; Zingl et al. 2020), is triggered by three factors: (i) the modulation of the cross-links in the envelope (OM–PG), (ii) the modification in the OM composition, and (iii) the turgor stress (Fig. 1A) (Bernadac et al. 1998; Hayashi et al. 2002; McBroom et al. 2006; Schwechheimer et al. 2014; Toyofuku et al. 2019; Zingl et al. 2020).

The cross-links in the envelope have been associated with vesicular biogenesis due to the role they play in the union and, in the integrity of the cell envelope in Gram-negative bacteria. Particularly, genes coding for the membrane porin OmpA in *Salmonella*, the lipoprotein NlpI and Tol-Pal system proteins in *Escherichia coli,* have been associated with vesicular biogenesis (Deatherage et al. 2009; Nevermann et al. 2019; Schwechheimer et al. 2014, 2015; Schwechheimer and Kuehn 2013). Disruptions in these genes prevent the formation of junctions between PG and OM, leading to the shedding of the OM in the form of OMVs (Deatherage et al. 2009; Schwechheimer and Kuehn 2015). In the same sense, the rearrangement or abundance of OM-PG bonds is regulated by small RNAs (sRNA), such as Reg26 and MicA, related to the down-expression of lipoproteins (Lpps) and OmpA, respectively (Choi et al. 2017; Schwechheimer et al. 2013; Song et al. 2008; Zingl et al. 2020).

The modification of components of the OM, such as the accumulation of phospholipids in *Haemophilus influenza* and *Vibrio cholerae*, or the introduction of molecules, such as the quinolone signal in *Pseudomonas aeruginosa* (Pseudomonas Quinolone Signal), alter the curvature of the membrane, promoting the release of OMVs (Florez et al. 2017; Gill et al. 2019; Roier et al. 2016; Toyofuku et al. 2019). Likewise, the structure of LPS, such as the deacylation of lipid A in *Salmonella typhimurium*, generates a differential curvature that results in the generation of OMVs (Elhenawy et al. 2016; Mozaheb and Mingeot-Leclercq 2020).

Turgor stress is the third factor related to the formation of OMVs by blebbing, arising from the accumulation of misfolded proteins, PG fragments, or LPS in the periplasm of Gram-negative bacteria (Ojima et al. 2016; Schwechheimer et al. 2014; Schwechheimer and Kuehn 2013). The abundant presence of these molecules increases the pressure on the cell wall, with the generation of OMVs being the mechanism to release intracellular stress to maintain cell homeostasis (Gill et al. 2019; Schwechheimer et al. 2014; Toyofuku et al. 2019).

In Gram-positive bacteria, BEV biogenesis can start with the budding or blebbing of CM (Fig. 1C) (Briaud and Carroll 2020; Jeong et al 2022). Lipidomic analyses of MVs have shown differences in phospholipid and fatty acid content between the vesicles and the CM (Briaud and Carroll 2020). MVs of *Listeria monocytogenes* present an abundance of phosphatidylethanolamine, triacylglycerols, and sphingolipids (Coelho et al. 2019), while MVs derived from *Lactobacillus plantarum* are abundant in phosphatidylcholine, diacylglycerol and lysophosphatidylserine (Kim et al. 2020a). This differential composition of lipids between vesicles and cells suggests that the production of MVs by Gram-positive bacteria could be linked to lipid domains enriched in the CM (Briaud and Carroll 2020).

In *Staphylococcus aureus,* the biogenesis of MVs can also be promoted by phenol-soluble modulins (PSMs) (Wang et al. 2018). The PSMs correspond to a family of amphipathic peptides with surfactant activity that cause local deformations in the CM associated with an increase in the turgor pressure of the cytoplasm, increases the curvature of the membrane, which detaches forming new MVs (Wang et al. 2020, 2018). The participation of Lpps in MV biogenesis in *S. aureus* has also been suggested (Wang et al. 2020).

The MVs outflow through the PG layer of Gram-positive bacteria has been related to PG-degrading enzymes or autolysins, observed in *S. aureus* or *Mycobacterium tuberculosis* (Briaud and Carroll 2020; Lee et al. 2009; Palacios et al. 2020; Prados-Rosales et al. 2011; Wang et al. 2021a, b). Additionally, the degree of links in the PG affects MV production (Briaud and Carroll 2020), as has been observed with *S. aureus* subjected to sublethal concentrations of penicillin G that decreases PG binding and increases MV release without affecting cell viability (Wang et al. 2018; Wyke et al. 1981).

### Explosive cell lysis and bubbling cell death

Cell lysis can lead to the biogenesis of diverse types of vesicles, OMVs, non-classical OMVs, or MVs, from Gram-negative membranes (Turnbull et al. 2016). Explosive cell lysis is triggered by the action of endolysins encoded in cryptic prophages, which are activated by DNA damage, and their role is to degrade PG *in P. aeruginosa* (Toyofuku et al. 2019; Turnbull et al. 2016). The BEV production mediated by endolysins in Gram-positive bacteria such as *Bacillus subtilis* and *Lactococcus lactis* has also been studied (Toyofuku et al. 2017; Liu et al. 2022a). In these bacteria, phage-encoded enzymes generate holes in the cell wall, allowing the release of the MVs, and although there is no explosive cell lysis, the cells die due to loss of cell integrity through the formation of bubbles, a process called bubbling cell death (Briaud and Carroll 2020; Toyofuku et al. 2017).

### Formation of nanotubes in the production of BEVs

The formation of filamentous structures called nanotubes has been found in a wide variety of bacteria, including Gram-positives (*B. subtilis* and *Clostridium acetobutylicum)* and Gram-negatives (*E. coli* and *Acinetobacter baylyi*) (Baidya et al. 2018)*.* The simplest morphology of the nanotubes is related to chains of OMVs, observed for the first time in cultures of *Myxococcus xanthus* (Remis et al. 2014; Toyofuku et al. 2019). These nanotubes participate in cell–cell communication and are considered a type of specialized BEVs (Baidya et al. 2018; Gill et al. 2019; Toyofuku et al. 2019). In Gram-positive bacteria, nanotube formation occurs through a process of extrusion of the plasma membrane through holes in the PG, while in Gram-negative bacteria, nanotubes are formed from the extrusion of the OM (Gill et al. 2019; Toyofuku et al. 2019) (Fig. 1).

## Biological functions of BEVs

**Fig. 2:**
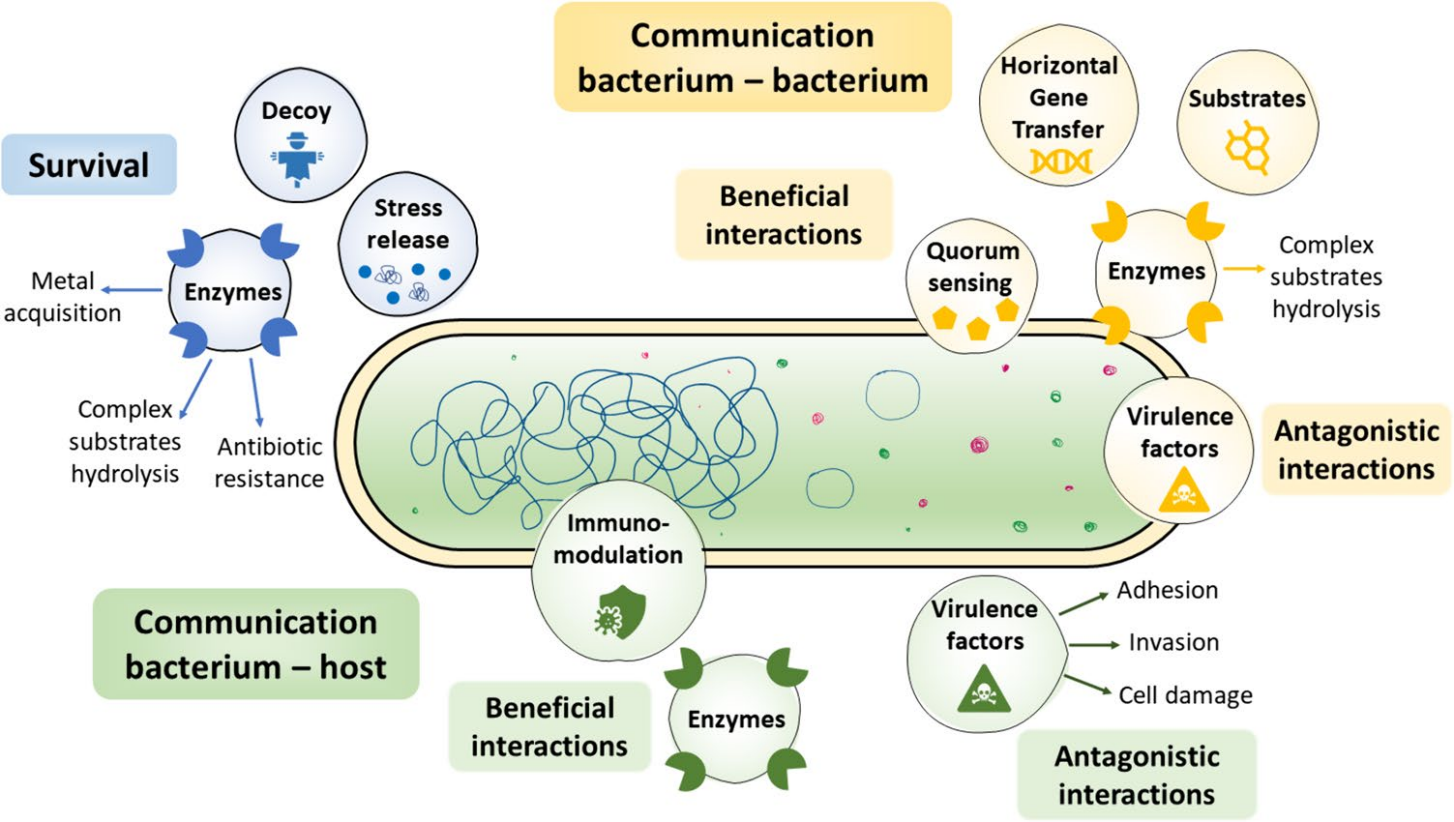
Biological functions of Bacterial Extracellular Vesicles (BEVs): bacterial survival, bacterium-bacterium communication, and bacterium-host communication. BEVs may act like decoys and release key enzymes involved in activities such as substrate degradation, metal acquisition, or antibiotic resistance. BEVs also help the cell release pressure. BEVs play beneficial or antagonistic roles during the communication process with other cells. They can transfer genes, enzymes, molecules to activate quorum sensing, and substrates to other bacteria. Also, BEVs could carry virulence factors to promote invasion by pathogenic bacteria

BEVs determine physiological functions in the life of the cells that produce them, despite their energetic or metabolic cost (Schwechheimer and Kuehn 2015) (Fig. 2).

### BEVs in the bacterial survival

In natural and artificial environments, bacteria are exposed to unfavorable biotic and/or abiotic factors, which affect their growth and viability (Guan et al. 2017; Mozaheb and Mingeot-Leclercq 2020). Whereas BEVs participate in molecular mechanisms that ensure the survival of bacteria, increasing their production (Guerrero-Mandujano et al. 2017; McBroom et al. 2006).

BEVs released by *Pseudomonas putida*, *E. coli,* or *V. cholerae* have been found to function as decoys in the presence of toxic or antimicrobial compounds such as toluene, polymyxin B, and colistin (Giacomucci et al. 2022; Kobayashi et al. 2000; Manning and Kuehn 2011). Another bacterial survival strategy against harmful compounds is loading vesicles with hydrolytic enzymes, such as β-lactamase in BEVs of *S. aureus* and *Moraxella catarrhalis,* conferring resistance to ampicillin and amoxicillin, respectively (Lee et al. 2013; Schaar et al. 2011). Bacteria like *Delftia acidovorans* and *Fibrobacter succinogenes* release OMVs and nanotubes, loaded with enzymes involved in the degradation of unconventional carbon sources such as phenanthrene, hemicellulose, and pectin, favoring bacterial survival in complex media (Arntzen et al. 2017; Shetty and Hickey 2014). Another function of BEVs is the acquisition of metallic elements, such as iron and zinc, essential for the survival of *Neisseria meningitidis* (Lappann et al. 2013; Schwechheimer and Kuehn 2015).

### BEVs in bacterium-bacterium interactions

BEVs are also used by bacteria as a communication mechanism with other bacteria, making these cell–cell interactions beneficial or antagonistic (Caruana and Walper 2020; Mozaheb and Mingeot-Leclercq 2020)**.** In bacterium-bacterium communication, BEVs might increase genetic diversity and modify the survival of other bacteria through horizontal gene transfer (Dell’Annunziata et al. 2021; Goreham et al. 2019; Kim et al. 2015a; Klieve et al. 2005; Schwechheimer and Kuehn 2015). Furthermore, BEVs disperse signals during quorum sensing in *P. aeruginosa* cultures (Caruana and Walper 2020; Lee et al. 2009; Lin et al. 2017) or participate as nucleation sites and in the transport of compounds during biofilms formation by *Streptococcus mutans* and *Shewanella vesiculosa* (Baeza and Mercade 2020; Begić and Josić 2020; Caruana and Walper 2020; Wang et al. 2015; Liao et al. 2014). BEVs released in a microbial community, generate other benefits, such as access to nutrients. *Bacteroides fragilis* and *Bacteroides thetaiotaomicron* release BEVs loaded with hydrolytic proteins, leads to the generation of molecules that are easily assimilated by other species of the same community (Caruana and Walper 2020; Carvalho et al. 2019; Elhenawy et al. 2014). Among the antagonistic functions of BEVs, the release of virulence factors and antimicrobial compounds are observed, affecting the viability of other bacteria (Caruana and Walper 2020; Tashiro et al. 2013). Outstanding examples are the inhibition of *E. coli* by BEVs of *Cytobacter velatus*, or the viability reduction of *Lactobacillus delbrueckii* by bacteriocin-loaded *Lactobacillus acidophilus* MVs (Dean et al. 2020; Schulz et al. 2018).

### BEVs in bacterium-host interactions

The study of communication between bacteria and eukaryotic cells through BEVs is another field of interest due to its influence on multiple diseases (Pathirana and Kaparakis-Liaskos 2016; Caruana and Walper 2020). Both, commensal and pathogenic bacteria can deliver effector molecules to mammalian cells, triggering cytotoxic, cytolytic, or immunomodulatory responses (Kim et al. 2015a; Pathirana and Kaparakis-Liaskos 2016). The type of response will depend on the origin of the BEVs (bacterial species), their concentration, and the target cell (Gurung et al. 2011; Kim et al. 2015a; Wang et al. 2021a, b). Generally, adverse responses are generated by vesicles of pathogenic bacteria (*E. coli*, *Shigella dysenteriae*,* V. cholerae*, *P. aeruginosa and S. aureus*), loaded with virulence factors, and their functions may correspond to invasion, adherence, damage to host cells or induction, of proinflammatory processes (Hu et al. 2020; Li et al. 2020; Wang et al. 2021a, b; Guerrero-Mandujano et al. 2017; Gurung et al. 2011). Meanwhile, BEVs from probiotic bacteria like *Lactobacillus* sp or *E. coli Nissle* 1917 carry effectors that protect the integrity of the intestinal epithelium against infections, maintain colorectal homeostasis during inflammation, and attenuate colitis or intestinal inflammation (Alvarez et al. 2019; Choi et al. 2020). In addition, BEVs of *Bacteroides thetaiotaomicron* transport enzymes that provide nutrients to the bacterium and remove carcinogenic metabolites from the host intestine (Carvalho et al. 2019; Goreham et al. 2019; Stentz et al. 2014). In plants, the activation of immune responses in the presence of BEVs from *Pseudomonas syringae* and *Pseudomonas fluorescens* protects them from pathogenic microorganisms such as other bacteria and oomycetes (McMillan et al. 2021).

## Biotechnological applications of BEVs and aspects to be resolved

**Fig. 3:**
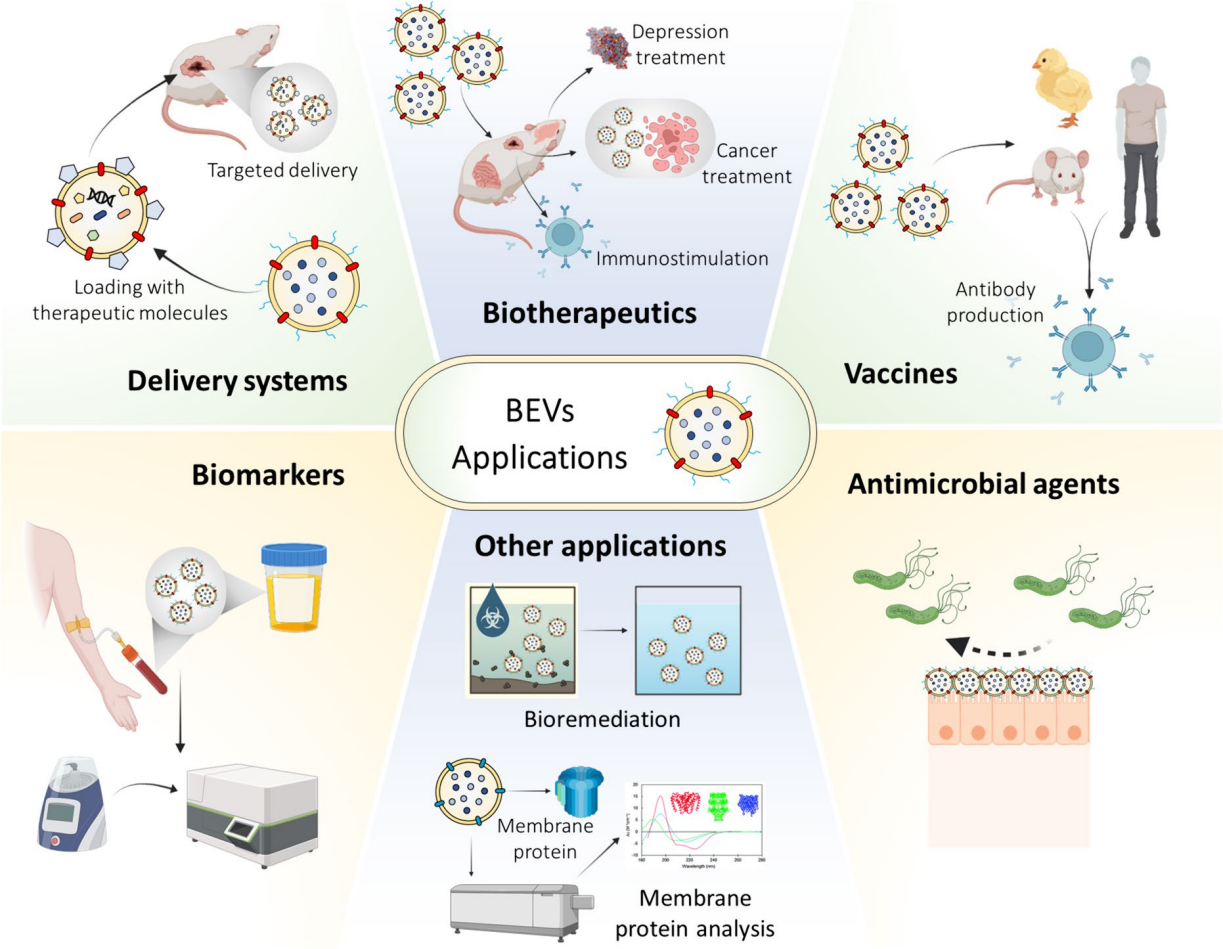
Bacterial Extracellular Vesicles (BEVs) applications. BEVs are biotherapeutics in the treatment of cancer, depression, or organ inflammation. BEVs as molecule transport systems. BEVs in the production of vaccines and antimicrobial compounds. BEVs as biomarkers, like tuberculosis screening. BEVs as a biotechnology tool for the study of lipid membrane permeability and membrane proteins. BEVs as nanoreactors in bioremediation or substrates enzymatic transformation. Created with Biorender.com

Biotechnological applications of BEVs have gained attention because of their interesting activities, such as containing bacterial antigens, pathogen-associated molecular patterns (PAMPs), intracellular communication cargo proteins, and immune system modulators (Huang et al. 2022a, 2022b; Li et al. 2020; Thoma et al. 2018). Among the biotechnological applications of BEVs (Table 1**, **Fig. 3), vaccine agents (van der Pol et al. 2015; Micoli and MacLennan 2020) and drug delivery systems have the greatest interest (Herrmann et al. 2021; Li et al. 2020).

**Table 1:** A representative sample of BEVs used in different biotechnological areas

Area	Use		Parental bacterium	Cargo		Target cells or organism	Effect of BEVs	References
Biotherapeutics	Immunotherapy. Formulation of new adjuvants		*Bifidobacterium longum*, *Clostridium butyricum*, and *Lactobacillus plantarum*	Peptidoglycan		Mouse macrophages (RAW264.7), Mouse dendritic cells (DC2.4)	Stimulation of the innate immune system. Production of proinflammatory cytokines TNF-α and IL-6. Acquisition of adaptive immunity. Cargo protection	Morishita et al. (2021)
	Cancer immunotherapy		*Escherichia coli.* Synthetic vesicles (SyBV) obtained from *E. coli* spheroplasts	–		Dendritic cells 3D spheroid melanoma (B16F10)	Activation of dendritic cells by SyBV. Using melanoma vesicles in conjunction with SyBV reduced tumor volume. In mice, inhibition of the growth of other types of cancer was observed	Park et al. (2021)
	Treatment of Inflammatory Bowel Disease Functional foods		*Lactobacillus kefir, L. kefiranofaciens,* y *L. kefirgranum*	*Lactobacillus* spp. molecules		Caco-2 cells Mice	Alleviation of inflammation induced by Tumor Necrosis Factor-α (TNF-α) in intestinal cells. Prevention of diarrhea and enterohaemorrhagic	Seo et al. (2018)
	Antidepressants		*Lactobacillus plantarum*	*L. plantarum* molecules		HT22 (mouse brain cells)	Increased expression of brain-derived neurotrophic factor (BDNF) and antidepressant effects	Choi et al. (2019)
	Treatment of breast cancer		*Escherichia coli* (DH5α)	Photosensitizer chlorin e6 and chemotherapeutic drug doxorubicin		Raw264.7 macrophages Mice	Triple-negative breast tumors (TNBC) eradication without side effects	Li et al. (2023)
	Treatment of colorectal cancer		*Lacticaseibacillus paracasei* PC-H1	Molecules derived from *L. paracasei*		Colorectal cancer cell line Mice	MVs inhibited proliferation and led to apoptosis of colorectal cancer cells (in vitro). MVs also promoted tumor apoptosis in an in vivo model	Shi et al. (2022)
Vaccines	Multiserotype vaccine against avian pathogenic *Escherichia coli* (APEC) based on OMVs (MOMVs)		*Escherichia coli* (APEC) serotypes O1, O2 and 78	OMPs (mainly OmpA), LPS		Macrophages (HD11) chicken	Activation of chicken macrophages. Production of antibodies in chicken vaccinated with MOMVs. High percentage of chicken survival	Hu et al. (2020)
	Induction of immune response against *Acinetobacter baumannii*		*Escherichia coli* transformed with Omp22	Membrane protein from *A. baumannii* Omp22		Mice	Increased survival rate of mice immunized with OMVs-Omp22 and inoculated with *A. baumannii*. Induction of specific antibodies. Bactericidal activity of OMVs-Omp22 against *A. baumannii* in experiments “in vitro”	Huang et al. (2016)
	Bexsero, vaccine against *Neisseria meningitidis* Group B		*Neisseria meningitidis*	Antigens: fHbp, NadA, NHBA, GNA1030 and 2091		Humans	Protection against meningococcus serotype B. BEVs enhance the activity of the adjuvant	Gorringe and Pajon (2012); Zanella et al. (2021)
	Candidate for an intranasal vaccine against SARS-CoV2		*Salmonella typhimurium*	Spike receptor-binding domain (RBD)		Hamster	BEVs elicit IgG production against Spike-RBD. Arouses neutralizing antibody activity against wild-type and Delta variants of the virus	Jiang et al. (2022)
	Generalized Modules for Membrane Antigens (GMMA) like vaccine against *Shigella sonnei*		*Shigella sonnei*	LPS with reduced Endotoxicity, immunodominant O-antigen		Mice and rabbits	Highly immunogenic. O antigen elicited substantial anti-LPS antibody levels	Gerke et al. (2015)
	GMMA like vaccine against meningococcal strains		*Salmonella typhimurium*	*Neisseria meningitidis* factor H binding protein (fHbp)		Mice	GMMA elicited antibodies against meningococcal strains, superior to the protein alone	Alfini et al. (2022)
“Delivery” systems	Anticancer: treatment of melanoma		*Escherichia coli* transformed with Tumor necrosis factor related ligand-induced apoptosis (TRAIL)	TRAIL, α_3_β_2_ integrin targeting ligand (protein overexpressed in invasive melanomas), inociadin green (loaded onto OMVs by fusion and electrostatic effects)		Melanoma spheroid 3D (B16F10)	Photothermal-photodynamic responses against primary spheroid melanomas (induced by NIR irradiation). Induction of apoptosis by TRAIL in disseminated tumor cells. Melanoma eradication	Peng et al. (2020)
	Drug delivery against cancer cells		*Escherichia coli* transformed with affibody specific for human epidermal growth factor receptor 2 (HER2.)	Affibody specific for HER2 (ligand) and siRNA against KSP (mRNA that is overexpressed in proliferative cells and tumors)		HER2-transfected NIH3T3 fibroblasts, HER2-overexpressing SKOV3 cells, HER2-negative NIH3T3, MDA-MB-231 mouse cells. Mice	Vesicle specificity and internalization in cells expressing HER2. Inhibition of the proliferation of lines that overexpress HER2. Regression of tumor growth in an animal model. No cytotoxicity of vesicles	Gujrati et al. (2014)
	Delivery of biologically active proteins to the gastrointestinal and respiratory tracts to protect against infection, tissue inflammation, and injury		*Bacteroides thetaiotaomicron* (Bt) transformed with antigens against *S. enterica* ser. enteric *typhimurium* and influenza A virus (IAV), and human protein	*S. enterica* antigens: St-OmpA, St-SseB IAV antigens: H-stalk protein H5 Human protein: keratinocyte growth factor 2 (KGF-2)		Mice	Induction of antigen-specific immune responses and antibodies production against *S. enterica* and IAV antigens. IAV protection. Reduction in infections severity and repair of the intestinal epithelium promoted by KGF-2	Carvalho et al. (2019)
Antimicrobial	Antimicrobial treatments	*Burkholderia thailandensis*			4-hydroxy-3-methyl-2-(2-non-enyl)-quinoline and long chain rhamnolipids	*Biofilm Streptococcus mutans* (Oral pathogen)	Biofilm integrity reduction Reduction of *S. mutans* viability	Yihui Wang et al. (2021a, b)
	Protection of stored foods and increase of shelf life	*Lactobacillus plantarum*			Molecules derived from* L. plantarum*	*Shewanella putrefaciens* (tuna spoilage cause)	Inhibition of *Shewanella putrefaciens* growth. Inhibition of oxidation reactions in tuna stored for five days at 4 °C	Lee et al. (2021)
Biomarkers	Tuberculosis diagnosis	*Mycobacterium tuberculosis*			Small RNAs: ASdes and MTB-miR5	–	qRT-PRC detection of ASdes and MTB-miR5 in vesicles from tuberculosis-infected patients	Lu et al. (2021)
Other biotechnology areas	Study of membrane proteins	*Escherichia coli*			OMPs: OmpG, FhuA, Tsx, BamA	–	OMVs maintain the unique amphipathic environment provided by lipid bilayers. The proteins under study retained their orientation, native structure, and functions	Thoma et al. (2018)
	Study of phospholipid permeability vesicle based	*Escherichia coli* BL21 DE3			Porins, LPS, other cargo molecules	–	Allows estimation of cell wall permeability of Gram-negative bacteria facilitated by porins. Tool for the identification of passive capture pathways of new compounds by bacteria	Richter et al. (2021a, b)
	Bioremediation	Phosphotriesterase (PTE)-transfected bacteria			Phosphotriesterase	–	BEVs maintain the enzymatic activity of PTE under different environmental conditions. Degradation of organophosphorus reagents in water and solid surfaces	Alves et al. (2018)
	Nano-scale bioreactors	*Escherichia coli* wt, *ΔtolA* and *ΔtolB*, transfected with photoactive decarboxylase (CvFAP) and/or hydratase (SmOhyA)			Photoactive decarboxylase and/or hydratase	–	OMVs protect enzyme stability and exhibit lipid decarboxylation and hydration activity Nano-reactors with multi-step biocatalytic reactions	Song et al. (2021)

The treatment of *N. meningitidis*, with VA-MENGOC-BC, MenBvac, and MeNZB, with a least 70% efficacy against meningitis caused by MenB (Holst et al. 2013), and the approval of BEXSERO vaccine points to the importance of the production of vesicles and the compounds they can transport (Holst et al. 2013). The advantages of BEVs are that they are non-replicative entities that mimic a part of a pathogen without causing disease (Ellis and Kuehn 2010). BEVs sizes allow their biodistribution through lymphatic vessels, as well as their uptake by different cells (Ellis and Kuehn 2010). Although vesicle components and size are variable, they are molecular entities that allow the presentation of antigens to the presenting reinforcement cells, and they have adjuvant properties that stimulate immune responses (Ellis and Kuehn 2010; Ellis et al. 2010; van der Pol et al. 2015). Furthermore, some BEVs are stable around 4–40 °C and against chemical treatments (Arigita et al. 2004). Even more, the polarization of the membranes can provide vesicle protector properties. Whereas LPS contained in OMV might act as an activator of immune cells, such as monocytes/macrophages, through TLR2 or TLR4/MD2 that could activate NF-κB and IRF3 expression and other proinflammatory molecules, which can also lead to high reactogenicity of the vaccines (Kovacs et al. 2011; Maisonneuve et al. 2014; Mancini et al. 2020). Other important molecules in BEVs are capsular polysaccharides (CPS) and LPS. CPS, combined with carrier proteins, might produce a good vaccine antigen (Middelton et al., 2017). Therefore, BEVs have been coupled with different antigens and glycoantigens to trigger immunological memory (Mancini et al. 2020).

Interestingly, the bioengineering of BEVs is a field that has broadened its spectrum of application, allowing for a selection of the charge and specific target and therefore the rational design of new therapies (Bitto and Kaparakis-Liaskos 2017; García-Manrique et al. 2018; Herrmann et al. 2021). It is highlighted that BEVs can be loaded with exogenous molecules such as antigens, ligands, enzymes, therapeutic proteins, nucleic acids molecules, among others, through genetic engineering, physical or chemical processes (Alves et al. 2018; Carvalho et al. 2019; Peng et al. 2020). Among bioengineered BEVs, the Generalized Modules for Membrane Antigens (GMMA) have been designed as delivery tools that can serve as carriers of polysaccharides or heterologous protein antigens (Mancini et al. 2021; Micoli et al. 2021). The GMMA are OM vesicles obtained from gram-negative bacteria genetically modified to eliminate endotoxins, which could cause reactogenicity in humans, and to obtain an over-vesiculating phenotype that improves yields (Di Benedetto et al. 2021; Hu et al. 2022; Micoli et al. 2021). GMMAs promise to be a vaccine design tool since they can induce strong immunogenicity, which can even be genetically manipulated to modulate systemic reactogenicity. These can induce strong immunogenicity due to the native presence of LPS, Lpps, and PGs (Hu et al. 2022; Mancini et al. 2021). Immunogenicity has been related to the size of the vesicles and their ability to present different antigens in a bacteria-like environment. However, the mechanisms of action are still being studied (Mancini et al. 2021). Interestingly, through genetic manipulation and loading strategies such as chemical conjugation, the risk of systemic reactivity of GMMA can be regulated, and functions for transporting polysaccharides or heterologous protein antigens can be conferred (Di Benedetto et al. 2021; Gerke et al. 2015; Mancini et al. 2021). GMMA production widely involves the growth of the hypervesiculating strain in a bioreactor and two sequential filtration processes (Gerke et al. 2015).

A variety of GMMA are in development, and some are in more advanced states, which have been tested in preclinical and clinical trials (Table 1) (Gerke et al. 2015; Mancini et al. 2021). GMMA have been tested as a platform for loading antigens such as hemagglutinin, associated with influenza A virus, or glycoproteins from rabies virus, enhancing humoral and antigen-specific cell-mediated responses in mice (Hu et al. 2022). Instead, the genetic modification of *Shigella sonnei* led to the production of GMMA with reduced endotoxicity LPS while maintaining the virulence plasmid associated with the O-antigen (Gerke et al. 2015). The GMMA from *S. sonnei* have been tested in phases I and II, and it is well tolerated after intramuscular, intranasal, and intradermal administration (Launay et al. 2017). Moreover, the vaccine based on GMMA derived from the engineered meningococcal B strain, which reduces CPS and LPS, has been tested in clinical trials, being safe and showing protection in volunteers (Keiser et al. 2010).

BEVs carrying therapeutic recombinant proteins have generated attention, especially if they can be directed to specific tissues. The fusion of proteins or molecules to the BEVs to be recognized by specific receptors in specific cells is a common strategy. For example, antigens can be directed to the periplasmic space to be packaged and released as BEVs (Kesty and Kuehn 2004; Muralinath et al. 2011). *Streptococcus* proteins have been fused with OmpA from the *E. coli* in the periplasm and successfully packaged into BEVs to induce the production of functional antibodies in immunized mice (Fantappiè et al. 2014). Furthermore, by employing bioconjugation systems or plug and display, the production of OMVs loaded with spike receptor binding domains (RBD) of SARS-CoV-2 and phosphotriesterases has been achieved (Alves et al. 2015; Jiang et al. 2022).

In recent studies, BEVs have been used to carry cancer-specific epitopes or non-coding RNAs (Grandi et al. 2018; Zhang et al. 2019a, b) to lasting antitumor immune response and might inhibit the growth of different tumoral cells (Chen et al. 2020a; Zhang et al. 2019a, b), being important in cancer immunotherapy (Zhang et al. 2019a, b). Similarly*, E. coli* BEVs loaded with chemotherapeutic drugs have been used to eradicate breast cancer cells in mice (Li et al. 2023). Moreover, *Lacticaseibacillus paracasei* and *L. plantarum* BEVs only loaded with endogenous cargo inhibited the proliferation of colorectal cancer and had antidepressant effects, respectively (Choi et al. 2019; Shi et al. 2022). These results open the doors to the development of new biotherapeutics.

BEVs have been tested as antimicrobial agents and biomarkers in other biotechnological areas. For example, *L. plantarum* MVs have been found to inhibit the growth of *Shewanella putrefaciens* in tuna (Lee et al. 2021), and *Helicobacter pylori* OMVs coated with nanoparticles of poly(lactic-co-glycolic acid), prevents the adhesion of *H. pylori* to epithelial cells (Zhang et al. 2019a, b). Tuberculosis could be diagnosed by detecting specific sRNAs, such as Asdes and MTB-miR5, encapsulated in M. tuberculosis MVs that could be obtained from blood samples of infected patients (Lu et al. 2021). Furthermore, BEVs can be used to support enzymes that degrade contaminating compounds (Alves et al. 2015; Thoma et al. 2018).

Around 802 clinical trials involving EVs (exosomes, ectosomes, microvesicles, or OMVs) have been registered at clinicaltrials.gov, of which only 48 are related to OMVs. Although using BEVs in various areas is promising, different limitations condition their application (Bitto and Kaparakis-Liaskos 2017). One of them is the heterogeneity in sizes and composition, which can bias the results in clinical trials (Alves et al. 2015; Bitto et al. 2017; García-Manrique et al. 2018). Another limitation is the presence of immunogens that can cause adverse effects, mainly when the purpose of BEVs is different from modulating immune responses (Balhuizen et al. 2021a; Bitto and Kaparakis-Liaskos 2017). Among the main concerns, the low production of BEVs become a critical factor for the development of therapeutics due to the high concentrations required to achieve efficiency in treatments and cover the demands (Balhuizen et al. 2021a; Hu et al. 2020; Jahromi and Fuhrmann 2021; Morishita et al. 2021; Reimer et al. 2021; van de Waterbeemd et al. 2013a). Further, more research is still needed on the use of vesicles as therapeutic agents, their toxicology, as well as on pharmacodynamics and pharmacokinetics of BEVs (García-Manrique et al. 2018; Jahromi and Fuhrmann 2021).

## Strategies to increase the productivity of BEVs

**Fig. 4:**
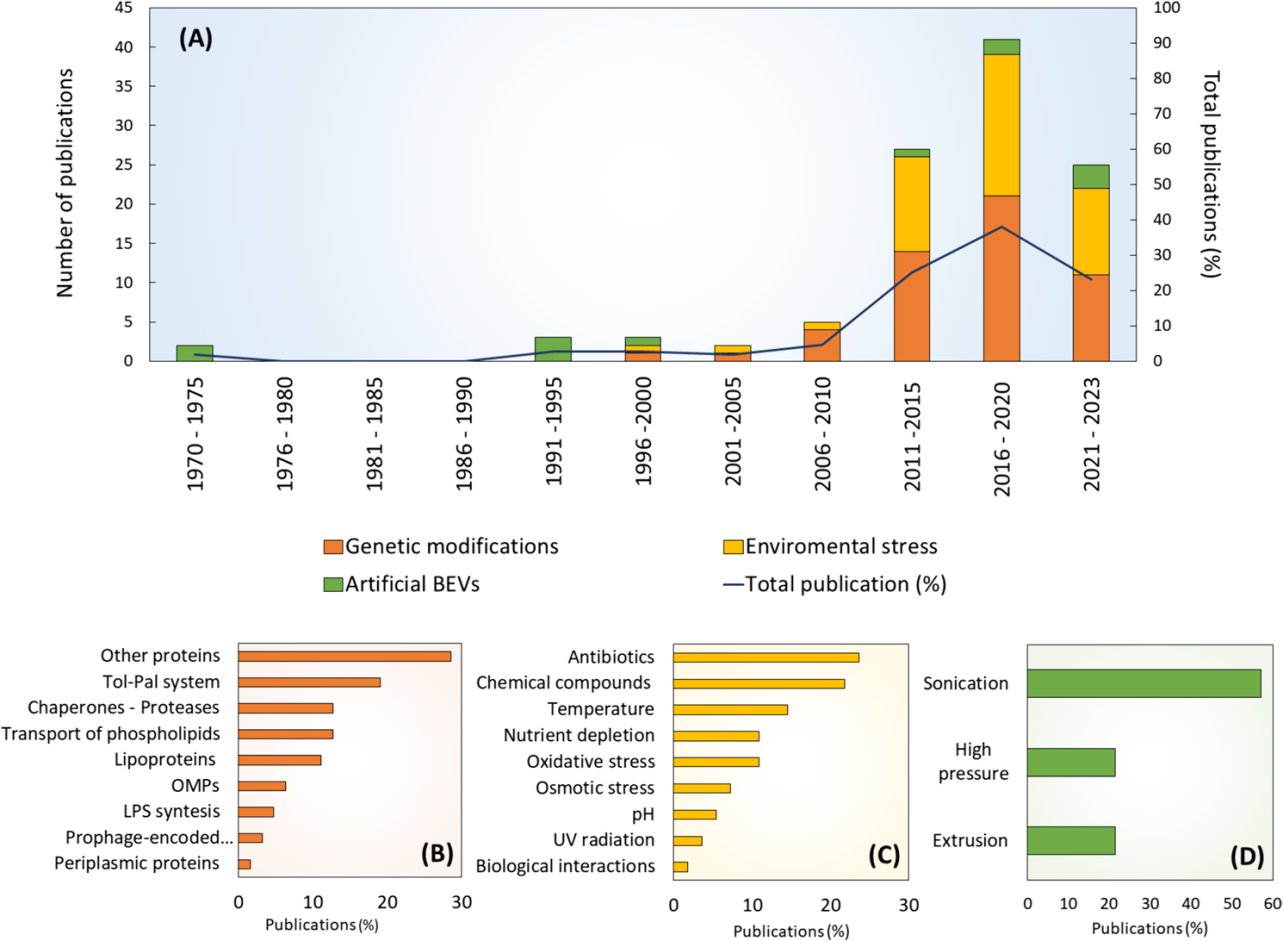
Strategies to increase the productivity of BEVs and updated bibliometric data on these strategies. (A) Bibliometrics data associated with abundant BEV production strategies over the last fifty years. (B) Publications report related to the genetic modifications of relevant proteins related to abundant vesiculation. (C) Publications report related to environmental and chemical stress conditions that lead to the abundant release of BEVs during bacteria culture. (D) Publications report related to methodologies employed by the artificial BEVs production

The productivity of EVs is typically low compared with the biomass, representing less than 1%. Different strategies to increase the productivity of BEVs have been explored over the last fifty years (Fig. 4) (Aytar-Çelik et al. 2022; Balhuizen et al. 2021a), such as genetic modifications of the cell envelope and culture conditions in stressful environments (Fig. 4B, C**, **Supplementary 1A, B, C)**.** An strategy used since the 1970s, is the production and recovery of artificial BEVs, highlighting the sonication (Balhuizen et al. 2021a) (Fig. 4D).

### Molecular strategies to increase BEVs formation: deletion, mutation, knock-out, or gene overexpression

Different genetic modifications have been evaluated as a strategy to increase BEV production, mostly based on the mutation, deletion, or knock-out of genes associated with proteins or processes of the cell envelope (Fig. 4B; Table 2) (Balhuizen et al. 2021a). One of the most used protein targets for developing strains with hypervesiculation is the Tol-Pal system (Pérez-Cruz et al. 2016; Reimer et al. 2021; Song et al. 2021; Takaki et al. 2020). This system contains a set of five distinct proteins that connect the IM with the PG and the OM, maintaining the structure of the cell envelope and participating in the cell division of Gram-negative bacteria (Mozaheb and Mingeot-Leclercq 2020; Zingl et al. 2020). TolQ, TolR and TolA form a complex in the IM and interact with TolB through TolA. TolB in the periplasm, binds to the Pal lipoprotein, which interacts with the PG via non-covalent bonds (Fig. S2) (Mozaheb and Mingeot-Leclercq 2020; Zingl et al. 2020). Defects in the components of this system lead to the formation of BEVs due to the dissociation of PG and CMs (Takaki et al. 2020).

**Table 2:** Increase in BEVs production by genetically modified bacteria, types of BEVs, and variations in physical, chemical, and biological characteristics

Bacterial strains	Genetic modification	Improved release of BEVs relative to wild-type strain	Measurement method	Characteristics of BEVs released by genetically modified strains	References
*Buttiauxella agrestis* JCM 1090^ T^	∆*tolB*	17-fold	FM4-64 assay	OMVs and M-OMVs with sizes between 20–150 nm and 400 nm, respectively	Takaki et al. (2020)
*Escherichia coli* K-12 BW25113	∆*tolA*	Ultracentrifugation (UC): twofold Ultradiafiltration (UF): fourfold	BCA assay, and malachite green phosphate assay	OMVs, O-IMVs, M-OMVs, G-OMVs with sizes: UC: 93–141 nm UF: 86–134 nm Presence of inner membrane proteins	Reimer et al. (2021)
*Escherichia coli* K-12 BW25113 (pUC19)	∆*tolA*	2.4-fold	FM4-64 assay	OMVs diameters from Δ*nlpI* were slightly less than that from WT, but those from Δ*rseA* and Δ*tolA* were similar to that of WT Δ*nlpI* enhances (threefold) the incorporation of plasmid in OMVs but in Δ*rseA* and Δ*tolA reduces*	Aktar et al. (2021)
	∆*rseA*	2.5-fold			
	∆*nlpl*	2.7-fold			
*Escherichia coli* BL21(DE3)	∆*tolA*	13-fold	Nanoparticle Tracking Analysis (NTA)	OMVs with sizes between 40 and 50 nm Abundant presence of OmpF and recombinant proteins CvFAP and/or SmOhyA	Song et al. (2021)
	∆*tolR*	21-fold			
*Escherichia coli* Nissle	∆*tolR*	52-fold	Purpald assay	OMVs, O-IMVs, M-OMVs, G-OMVs, with sizes between 20 and 200 nm Flagellin and MipA decrease Reduction in the internalization capacity of vesicles ∆*tolR* in host cells	Pérez-Cruz et al. (2016)
*Helicobacter pylori* 26,695	∆*tolB*	600-fold	ELISA	BEVs most immunogenic	Turner et al. (2015)
	∆*pal*	22-fold	ELISA	**–**	Turner et al. (2015)
*Staphylococcus aureus* JE2	∆*lgt*	4-fold	NTA	MVs with sizes around 60 nm Decrease in the content of pore-forming proteins (PFTs) Cells showed an increase in membrane fluidity	Wang et al. (2020)
*Staphylococcus aureus* Newman	∆*lgt*	2-fold	NTA	**–**	(Wang et al. 2020)
*Escherichia coli K-12 BW25113*	∆*nlpI*	6-fold	Densitometry of OMPs	Increased amount of cargo molecules (recombinant GFP)	Ojima et al. (2018)
*Escherichia coli* Nissle	∆*nlpI*	2-fold	–	Spherical OMVs with sizes between 80 and 400 nm	Thomas et al. (2022)
*Escherichia coli K-12 BW25113*	∆*degP*	8-fold	Densitometry of OMPs	–	Ojima et al. (2018)
*Escherichia coli K-12 BW25113*	∆*mlaE*∆*nlpI*	30-fold	Densitometry of OMPs	OMVs, O-IMVs, M-OMVs, with sizes around 100 nm Increased amount of cargo molecules (recombinant GFP)	Ojima et al. (2020, 2021)
*Neisseria meningitidis*	*ΔrmpM*	3-fold	Protein content	–	Maharjan et al. (2016)
*Escherichia coli* ULS153	Deletion operon Dlm	4-fold	Densitometry of OMPs	–	Pasqua et al. (2021)
*Buttiauxella agrestis* JCM 1090^ T^	∆*ompA*	13-fold	FM4-64 assay	–	Takaki et al. (2020)
*E. coli enterohemorrhagic* (EHEC)	Plasmid insertion pEHompT	40-fold	Transmission electron microscopy (TEM)	OMVs with sizes up to 20 nm Reduction in protein and lipid content	Premjani et al. (2014)

The deletion of the *tolB* gene in the *B. agrestis* JCM 1090 T increased the release of vesicles 17-fold compared to the wild strain, promoting BEV formation from the poles of the cell, at the sites of cell division and on the lateral surfaces (Takaki et al. 2020). Similarly, the *tolA* modification in *E. coli* K-12 BW25113 and *E. coli* BL21(DE3) increased the release of BEVs four and 13-fold, respectively, relative to wild-type strains (Reimer et al. 2021; Song et al. 2021). The *tolR* mutation of *E. coli* Nissle 1917 increased BEV production to 52-fold compared to the wild strain (Pérez-Cruz et al. 2016), also causing differences in the protein composition, and heterogeneity in size and shape of BEVs (Pérez-Cruz et al. 2016). The physical heterogeneity was derived in a loss in the interaction capacity of the BEVs of *E. coli* Nissle 1917 with epithelial cells (Pérez-Cruz et al. 2016). Interestingly, the variability in size and morphology in BEVs also has been observed in *E. coli* K-12 BW25113 *ΔtolA* and *B. agrestis ΔtolB* strains, obtaining O-IMVs, multilamellar vesicles (M-OMVs), multivesicular (G-OMVs), and partially circularized vesicles (Pérez-Cruz et al. 2016; Reimer et al. 2021; Takaki et al. 2020).

Another group of key genes for generating bacteria with hypervesiculation are those associated with the synthesis of Lpps (Schwechheimer et al. 2014, 2015; Schwechheimer and Kuehn 2013). The Lpps, also known as cross-linking proteins, correspond to peripheral membrane proteins with a hydrophobic tail that serves as an anchor to the lipid bilayer (Mathelié-Guinlet et al. 2020; Mozaheb and Mingeot-Leclercq 2020). In Gram-positive bacteria, Lpps are further attached to the cytoplasm via an N-terminal lipid residue, and their functions include maintaining the integrity and organization of the CM (Wang et al. 2020; Wang et al. 2021a, b). The mutation of the *lgt* gene coding for the lipoprotein diacylglycerol transferase increased the fluidity of the plasmatic membrane, doubling or tripling MVs production by the *S. aureus* JE2 *Δlgt* and *S. aureus* Newman *Δlgt* strains with respect to wild-type strains (Wang et al. 2020). The BEVs released from mutated bacteria presented smaller sizes and alterations in protein content, such as a reduction of Pore-forming toxins (PFTs) used by *S. aureus* as virulence factors (Wang et al. 2020). In Gram-negative bacteria, Lpps are anchored to PG via a C-terminal lysine (Zingl et al. 2020). Mutation of the *nlpI* gene encoding the OM Lpp NlpI in *E. coli* Nissle 1917 and *E. coli* K-12 BW25113 increased vesiculation two-fold and six-fold, respectively, compared to wild-type strains (Ojima et al. 2018; Thomas et al. 2022). NlpI participates in cell division and in the negative regulation of PG endopeptidases, and its mutation reduces the crosslinking of Lpp and PG by up to 40%, increasing indirectly the formation of BEVs (Ojima et al. 2018; Schwechheimer et al. 2015).

On the other hand, deletion of the *degP* increased the production of OMVs up to eight-fold without affecting growth of *E. coli*. The *degP* codes for a periplasmic chaperone-protease that removes misfolded proteins in the cell envelope (Ojima et al. 2018; Schwechheimer et al. 2015). The mutation of *degP* leads to the accumulation of proteins in the cell wall, generating cellular stress and causing the production of BEVs to release accumulated proteins (Ojima et al. 2018; Schwechheimer and Kuehn 2013).

Interestingly, in BEVs from *E. coli ΔnlpI* and *E. coli ΔdegP* the loading with recombinant protein OmpW-GFP (GFP, green fluorescent protein) is differential, being higher in vesicles from *E. coli ΔnlpI* (Ojima et al. 2018). This suggests the importance of hypervesiculation and the incorporation of cargo molecules.

In wild strains of Gram-negative bacteria, the *vacJ* (*mlaA* in *E. coli*), in conjunction with *ybr* or *mla* (*B, C, D, E* and *F),* encode for subunits of the ABC transporter VacJ/Yrb, whose function is to maintain the OM asymmetry (Malinverni and Silhavy 2009; Roier et al. 2016). Studies on the biogenesis of OMVs in *Haemophilus influenzae* and *V. cholerae* revealed that the deletion or reduction in the expression of the *vacJ* and *yrb* cause the accumulation of phospholipids in the outer leaflet of the OM. To maintain membrane asymmetry, i.e., LPS in the outer leaflet and phospholipids in the inner leaflet, bacteria secrete phospholipids through OMVs (Roier et al. 2016).

Double mutants of *E. coli* K-12 BW25113 (*ΔmlaE*:*ΔnlpI*) showed an increase of BEVs by 30-fold when compared with the wild-type strain, where the release of vesicles was associated with the reduction of cross-links between PG and Lpp, as well as with the accumulation of phospholipids in the cell OM (Ojima et al. 2020, 2021). In addition, the mutant *E. coli ΔmlaE:ΔnlpI* presented elongated cells, and BEVs generated mainly from the poles of the cells with sizes larger compared to the wild-type strain (Ojima et al. 2021). While deleting the lysis module (Dlm) in *E. coli* ULS153 increased up to fourfold the production of OMVs (Pasqua et al. 2021). Dlm consists of an operon of four genes (*essD, ybcS* and *rzpD/rzoD*) encoding a holin (S), an endolysin (R), and two spanins (Rz/Rz1), which contribute to the release of PG residues (Pasqua et al. 2021). The holin generates small holes in the internal CM for the passage to the periplasm of endolysin and spanins, which are responsible for causing cell lysis (Pasqua et al. 2021; Turnbull et al. 2016).

In addition, another mechanism for the abundant production of BEVs is the overexpression of the OmpT protease (Premjani et al. 2014). In Enterohemorrhagic *E. coli* (EHEC), this modification released 40-fold more OMVs than the wild-type strain (Premjani et al. 2014). It is considered that high levels of OmpT in the OM altered the number of contacts between the PG and the OM via proteolysis, leading to membrane detachment and the formation of BEVs (Premjani et al. 2014). The overproduction of the OmpT protease impacts the characteristics of the OMVs, which have smaller diameters, and lower protein and lipid content compared to the OMVs of the wild-type strain (Premjani et al. 2014).

In general, from the productive point of view, the modifications in the *tolB* and *tolR* genes of the Tol-Pal system, and the double knock-out of the *nlpI* and *mlaE* genes, are the most promising systems to obtain strains with hypervesiculation (Fig. 4B; Table 2). However, although genetic modification makes it possible to obtain strains that overproduce BEVs, these are heterogeneous in size, composition, and morphology, so it is necessary to evaluate the advantages and disadvantages in the development of new biotherapeutics, vaccines, and delivery systems.

### Culture strategies to obtain abundant BEVs

Throughout evolution, bacteria have acquired various adaptation mechanisms (Guan et al. 2017; Hews et al. 2019), including the ability to sense environmental changes and use them as inducers of various cellular responses to stress (Hews et al. 2019). The cell envelope is the first line of defense of bacteria, so there are specific envelope stress response systems (ESRS) that ensure cellular homeostasis, maintain its integrity and fluidity (Eberlein et al. 2018; Guan et al. 2017), and sense extracellular stress and perturbations in the periplasm (Hews et al. 2019; Laloux and Collet 2017).

The release of BEVs has been cataloged as a stress response system in the envelope (Klimentová and Stulík 2015; McBroom and Kuehn 2007; Mozaheb and Mingeot-Leclercq 2020), which maintains the composition of the periplasm in balance through selective packaging and secretion of harmful material (McBroom and Kuehn 2007). This ESRS may act as an immediate response mechanism protecting the OM (Eberlein et al. 2018; Manning and Kuehn 2011). Vesiculation involves the extraction of misfolded proteins from the cell, changes in the composition of the OM, or neutralize the deleterious effect of toxic compounds, allowing the bacteria activate ESRS with complex signaling pathways (Eberlein et al. 2018; Manning and Kuehn 2011; Mozaheb and Mingeot-Leclercq 2020). Bacteria also use the release of BEVs to export regulatory proteins and proteolytic products accumulated in the envelope derived from other ESRS (McBroom and Kuehn 2007).

Since, bacteria respond to external stimuli by modulating the composition of the cell envelope and producing BEVs (Klimentova et al. 2019; Klimentová and Stulík 2015; Yokoyama et al. 2021), stressful conditions has been used to increase vesiculation (Klimentová and Stulík 2015; Wang et al. 2021a, b). Stress factors, evaluated as strategies to increase the release of BEVs, are divided into two categories, environmental stress, and chemical stress (Fig. 4C and Table 3).

**Table 3:** Environmental stress factors that are associated with the abundant production of BEVs

Stress factor	Effector	Bacterial strain	Improved release of BEVs relative to control	Measurement method	Characteristics of BEVs released under stressful conditions relative to control	Reference
pH	pH (5.5)	*Streptococcus mutans*	10-fold	BCA assay	Smaller MVs (105 ± 11.6 nm) vs control (129 ± 8.08 nm). Reduction and differences in proteins	Cao et al. (2020)
	pH (5.3)	*Francisella tularensis*	3-fold	FM1-43 assay	Spherical and nanotubes OMVs. Reduction of proteins related to O-antigen, lipid A, phospholipid, and fatty acid biosynthesis	Klimentová et al. (2019)
	pH (5.8) and Mg^2+^ (10 µM)	*Salmonella enterica*	6-fold	FM4-64 assay	Larger OMVs (68 ± 5.67 nm) relative to control (43 ± 2,63 nm)**.** Increase in the amount of acylated lipids A	Bonnington and Kuehn (2016)
Temperature	Thermal shock: (56 °C / 60 min) Verwey medium	*Bordetella pertussis*	3.5-fold	Purpald assay	Spherical OMVs, diameters between 10 and 80 nm. Protein patterns like control OMVs, with high protein concentration	Balhuizen et al. (2021b); De Jonge et al. (2021)
		*Bordetella bronchiseptica*	18-fold	Purpald assay	Spherical OMVs, diameters between 15 and 40 nm. Increased protein concentration, phosphatidylglycerol and lysophospholipids, and reduction of phosphatidylethanolamine	
	Culture at low temperature (25 °C)	*Francisella tularensis*	0.63-fold	FM1-43 assay	Spherical OMVs with different sizes, and a low proportion of nanotubes. High level of proteins associated with biosynthesis of O-antigen and lipid A. Decrease in proteins associated with LPS and phospholipid transport	Klimentova et al. (2019)
	Culture at high temperature (42 °C)	*Francisella tularensis*	4.75-fold	FM1-43 assay	OMVs as long nanotubes, and low proportion of spherical particles. Different protein patterns compared to the control. Reduced proteins related to O-antigen and lipid A biosynthesis	Klimentova et al. (2019)
	Culture at low temperature (30 °C)	*Staphylococcus aureus*	2.9-fold	Bradford assay	MVs with increased in the LTA content	Wang et al. (2021a, b)
Oxidative stress	Culture with H_2_O_2_ (1.0 mM)	*Staphylococcus aureus*	1.7-fold	Bradford assay	MVs with an increase in the amount of α-hemolysin, and leukocidin	Wang et al. (2021a, b)
	H_2_O_2_ addition (5.0 mM; 10.0 mM)	*Francisella tularensis*	0.15 and 0.34-fold	FM1-43 assay	OMVs with morphology and composition like the control	Klimentova et al. (2019)
	Increased TOD in continuous culture (150%)	*Neisseria meningiditis*	4-fold	FM 4–64 assay NTA	Larger OMVs (110 nm) regarding to control (80 nm)	Gerritzen et al. (2018)
	Increased TOD in Batch culture (100%)	*Neisseria meningiditis*	3-fold	FM 4–64 assay	Larger OMVs (90 nm) vs control (80 nm). Minimal production of OMVs during exponential growth. Vesiculation increased as cysteine was depleted, being necessary against oxidative stress	Gerritzen et al. (2018)
	Increased DOT: Atmosphere of 15% O_2_ and 6% CO_2_ Control condition: Atmosphere of 6% de O_2_, 10% de CO_2_ and N_2_ 85%	*Campylobacter jejuni* Microaerobic	≈ 4.7-fold	BCA assay	OMVs enriched with PorA (changes the membrane permeability), flagellar protein A (accumulated during membrane destabilization), and Cjj81176_021 (periplasmic iron-binding protein)	Godlewska et al. (2019)
	Culture under anoxic conditions	*Pseudomona aeruginosa*	6-fold	FM 4–64 asssay	OMV with sizes between 20 and 90 nm. Presence of OMP. Enrichment with GroEL and pyocin assembly-related proteins	Toyofuku et al. (2014)
Osmotic stress	Exposure to NaCl (0.5 M / 30 min)	*Listeria monocytogenes*	≈ 1.2-fold	Bradford assay	Spherical MVs with sizes between 20 and 100 nm. Higher protein content vs control. Presence of virulence-related proteins (LLO and InlB), osmotic stress related proteins (GbuA), factor σ^B^-dependent proteins, RecA and UvrA	Lee et al. (2018)
Nutrient depletion	Low cysteine concentration	*Neisseria meningiditis*	12.45-fold	FM 4–64 assay	OMVs with sizes around 97 ± 9 nm. High amounts of PorA antigen	van de Waterbeemd et al. (2013a, b)
	Low sulfate concentration	*Neisseria meningiditis*	2.3-fold	NTA	OMVs with sizes around 87 nm, like OMVs at low cysteine concentration. OMVs enriched in phospholipids and low LPS vs OMS formed under low cysteine	Gerritzen et al. (2019a)
	Low iron concentration	*Staphylococcus aureus*	2.7-fold	Bradford assay	MVs with α-hemolysin, leukocidins and LTA. Pore-forming proteins (hemolysin-α and leukocidins) probably to promote iron acquisition	Wang et al. (2021a, b)
Ultraviolet radiation	Exposure to UV radiation for 6 h	*Cylindrospermopsis raciborskii*	2.2-fold	TEM	Spherical OMVs with larger sizes (99.54 ± 4.53 nm) vs control (86.23 ± 4.86 nm). OMVs covered by extracellular polymeric substances (EPS). The EPS could ensure the structural integrity of cells. Phosphatidylserine re-localization associated with OMVs release	Zarantonello et al. (2018)
	Exposure to UV radiation for 3 h	Non-axenic bacteria of freshwater	2.2-fold	TEM	Spherical OMVs with smaller diameters (45.26 ± 3.90 nm) vs control (78.77 ± 6.00 nm). OMVs participate in the homeostasis of the aquatic microbiota	Gamalier et al. (2017)
Biological stress	Interaction with* Microcystis aeruginosa*	*Cylindrospermopsis raciborskii*	1.8-fold	TEM	Spherical OMVs with larger sizes (101.50 ± 4.62 nm) compared to control (86.23 ± 4.86 nm). OMVs covered by extracellular polymeric substances (EPS)	Zarantonello et al. (2018)
Chemical compounds	Ethanol (1%)	*Staphylococcus aureus*	2.5-fold	BioRad protein assay	MVs with increased in the LTA content	Wang et al. (2021a, b)
	D-cycloserine (250 µg/L):	*Pseudomonas aeruginosa*	9.2-fold	FM4-64 assay		MacDonald and Kuehn (2013)
	Glycine 1.0% in LB broth	*Escherichia coli*—Nissle 1917	69-fold	Bradford assay	Glycine-induced MVs mean diameter (36.3 ± 15.0 nm) was significantly greater than that of non-induced MVs (28.2 ± 9.54 nm). No significant difference in the ratio of abnormal MVs to total MVs. The protein profile of glycine-induced MVs was similar to that of non-induced MVs	Hirayama and Nakao (2020)
			51-fold	FM4-64 assay		
			8.1-fold	Limulus assay		
	Glycine 1.0% in LB broth	*Escherichia coli* BW25113/pUC19	32-fold	FM4-64 assay	Glycine enhances by 13-fold the incorporation of plasmid in OMVs. Glycine increases the membrane permeability Changes in size distribution of OMVs were observed	Aktar et al. (2021)
	Glycine C1: 5 g/L C2: 10 g/L C3: 15 g/L C4: 20 g/L	*Limosilactobacillus antri* JCM 15950	C1: 5-fold C2: 8-fold C3: 12-fold C4: 25-fold	FM4-64 assay	Glycine > 15 g/L reduces bacterial growth by 50% Timing of glycine addition on MV production was important Glycine-induced MVs promoted immunostimulatory activity that was comparable to that of spontaneously produced MVs	Yamasaki-Yashiki et al. (2024)
	Lysine C1: 1.3 g/L C2: 2.6 g/L Control: 0.26 g/L	*Shewanella vesiculosa*	C1: 1.7-fold C2: 4.3-fold	FM4-64 assay	O-IMVs with diameters between 100 and 120 nm. Presence of H1275, a sensor protein involved in the production of O-IMVs and in the regulation of biofilms. O-IMVs could transport H1275 to other cells to regulate biofilm formation	Yokoyama et al. (2021)
	Sucrose fatty acid ester (SFE) C1: 10.0 µg/mL C2: 20.0 µg/mL C3: 30.0 µg/mL C4: 40.0 µg/mL	*Bacillus subtilis*	C1: 3.5-fold C2: 5.5-fold C3: 7.0-fold C4: 12-fold	FM1-43 assay	Spherical MVs with size of 117.8 ± 7.2 nm. Presence of the autolysin LytC and SFE in MVs. MVs more flexible or unstable. MVs increased the survival of *B. subtilis* above 90%, cultured with 40 µg/mL of SFE	Abe et al. (2021)
Antibiotics	Gentamicin C1: 0.1 µg/L C2: 0.3 µg/L	*Acinetobacter baylyi*	3-fold	Quant-iT PicoGreen dsDNA assay	Spherical OMVs with larger diameters (C1: up to 349 nm; C2: up to 389 nm) vs control (up to 304 nm). OMVs with low Z potentials (27.6 mV) vs control (− 16.7 mV)	Fulsundar et al. (2014)
	Polymyxin B (5.5 µg/mL)	*Campylobacter jejuni*	≈ 5.9-fold	BCA assay	OMVs with an increase in PorA and Cj1613c proteins Reduction of HtrA serine protease	Godlewska et al. (2019)
	Ciprofloxacin (CIP): 30.0 ng/mL Chloramphenicol (CF): 2.0 µg/mL	*Escherichia coli* *∆ompA*	CIP: 2.5-fold CF: 1.25-fold	FM1-43 assay	In the presence of antibiotics, BEVs trap toxic molecules, discard damaged cellular components, or send signals to other cells	Bos et al. (2021)
	Ampicillin C1: 16.0 µg/mL C2: 64.0 µg/mL	*Staphylococcus aureus*	C1: 9-fold C2: 22.4-fold	BCA assay	Spherical MVs with smaller sizes (78.22 ± 0.81 nm) vs control (86.84 ± 0.25 nm). Low Z potential values (− 30 mV). Increased in protein concentration. Increased proteins related to the degradation of β-lactam antibiotics, including β-lactamase, increasing the bacteria survival	Kim et al. (2020a, b)
	Cathelicidin PMAP-36 (0.5 µM)	*Bordetella bronchiseptica*	1.7-fold	FM4-64 assay	Spherical and tubular OMVs with average sizes between 20 and 40 nm. Increase in LPS and phosphatidylglycerol. Reduction in lysophosphophatidylglycerol	Balhuizen et al. (2021b)

#### Cultivation at acidic pH

The cultivation of *S. mutans*, *Salmonella enterica*, and *Francisella tularensis* at pH between 5.3—5.8, increases the number of BEVs up to tenfold higher than cultures at neutral pH (Table 3) (Bonnington and Kuehn 2016; Cao et al. 2020; Klimentova et al. 2019).

The size and morphology of BEVs produced in acidic environments vary according to the bacteria; for example, the *S. mutans* MVs were smaller at pH 5.5 than 7.5 (Cao et al. 2020). The modification of the pH and reduction in the concentration of Mg^2+^ (pH 5.8 and 10 μM of Mg^2+^), leads to the release of BEVs 20 nm larger by *S. enterica*, compared to the control (pH 7.6 and 10 mM Mg^2+^) (Bonnington and Kuehn 2016). Meanwhile, *F. tularensis* releases OMVs in the form of nanotubes at pH 5.3 (Klimentova et al. 2019), which are used by bacteria as connection bridges with other cells (Gill et al. 2019; Klimentova et al. 2019).

The MVs obtained from *S. mutans* at acidic pH presented fewer proteins, and ABC transporters were identified as the most important transport pathways during the bacteria growth under stress conditions (Cao et al. 2020). The reduction in the number of proteins in the MVs of *S. mutans* was attributed to these transporters (Cao et al. 2020). Likewise, the OMVs of *F. tularensis* show a reduction in the concentration of proteins associated with the biosynthesis of O-antigen, lipid A, phospholipids, and fatty acids, reflecting changes in the protein and lipid composition of the OM in response to environmental variation (Klimentova et al. 2019).

#### Cultivation at low and high temperatures

Thermal stress is another factor that increases BEV production (Klimentová and Stulík 2015; Mozaheb and Mingeot-Leclercq 2020). When the temperature varies, different cellular components suffer alterations, like the accumulation of misfolded proteins and modifications in the acyl chains of phospholipids, which are among the most common (Eberlein et al. 2018; Klimentová and Stulík 2015; Schwechheimer et al. 2013).

Vesiculation modulated by thermal stress has been evaluated in *Bordetella pertussis*, *Bordetella bronchiseptica*, *F. tularensis*, *P. putida*, *P. aeruginosa*, *A. baylyi* and *S. aureus*, with increases in the number of vesicles up to 39-fold compared to control conditions (Table 3) (Baumgarten et al. 2012; De Jonge et al. 2021; Fulsundar et al. 2014; Klimentova et al. 2019; MacDonald and Kuehn 2013; Wang et al. 2021a, b). Although hypervesiculation is frequent in cultures with temperature increases, it has been observed that strains such as *S. aureus* release a greater number of vesicles below 30 °C (Wang et al. 2021a, b). BEVs derived from the cultivation of bacteria under thermal stress present spherical morphologies (Balhuizen et al. 2021b; Baumgarten et al. 2012; De Jonge et al. 2021). However, *F. tularensis*, for example, secretes long tubular BEVs when grown at 42 °C, and spherical BEVs of heterogeneous sizes and nanotubes when grown at 25 °C (Klimentova et al. 2019).

BEV composition changes according to the bacterium and heat shock condition (Balhuizen et al. 2021b; De Jonge et al. 2021; Klimentova et al. 2019; Wang et al. 2021a, b). The inactivation by heat shock of *B. pertussis* and *B. bronchiseptica* resulted in OMVs with protein patterns like the controls, but a higher amount of protein, an increase in phosphatidylglycerol and lysophospholipids, and a reduction in phosphatidylethanolamine was found. The increase in lysophospholipids, characterized by having a single fatty acid, could also lead to a decrease in stability in the BEVs of *B. pertussis* and *B. bronchiseptica* (Balhuizen et al. 2021b). On the other hand, the culture of *F. tularensis* at 25 °C and 42 °C generated BEVs with protein compositions different from each other and from the control (Klimentova et al. 2019). For *S. aureus* the reduction in temperature favored the incorporation of LTA in the MVs (Wang et al. 2021a, b).

#### Cultivation under oxidative stress

Oxidative stress is a condition associated with the accumulation of reactive oxygen and reactive nitrogen species in cells. It causes damage to DNA, membrane lipids, and proteins and results in cell death (Ezraty et al. 2017). As defense systems, bacteria synthesize neutralizing enzymes of reactive species, activate stress responses, and release BEVs (Ezraty et al. 2017; Mozaheb and Mingeot-Leclercq 2020).

The contact of *S. aureus*, *F. tularensis,* and *P. aeruginosa* with sublethal concentrations of hydrogen peroxide (H_2_O_2_) or ciprofloxacin, the increase in dissolved oxygen tension in cultures of *N. meningiditis* and *Campylobacter jejuni*, and the growth of *P. aeruginosa* in denitrifying and anoxic conditions, triggered the abundant production of BEVs, up to six-fold higher than controls (Table 3) (Gerritzen et al. 2018; Godlewska et al. 2019; Klimentova et al. 2019; Toyofuku et al. 2014; Wang et al. 2021a, b). The abundant vesiculation could be associated with the accumulation of misfolded proteins in the periplasm due to the presence of H_2_O_2_ (MacDonald and Kuehn 2013), or to the synthesis of pyocins in anoxic conditions, which might induce disruptions in the IM, PG, and OM links (Toyofuku et al. 2014). The morphology of BEVs produced under oxidative stress depends on the parental bacterium, having OMVs in the form of nanotubes from *F. tularensis* (Klimentova et al. 2019), and spherical vesicles with diameters between 20 and 110 nm, from *P. aeruginosa*, *S. aureus*, *N. meningitidis*, and *C. jejuni* (Gerritzen et al. 2018; Godlewska et al. 2019; Toyofuku et al. 2014; Wang et al. 2021a, b).

BEV composition changes according to oxidative stress type and bacteria. The H_2_O_2_ changes the composition of the LPS in OMVs of *P. aeruginosa* (MacDonald and Kuehn 2013). While ciprofloxacin reduces the amount of LTA in MVs in *S. aureus* (Wang et al. 2021a, b). *C. jenuni* culture with a high TOD released OMVs loaded with PorA, OMP that alters the permeability of the CM, and Cjj81176_0211, an iron-binding protein (Godlewska et al. 2019). Whereas *P. aeruginosa,* under anoxic conditions, released vesicles enriched with the chaperone GroEL and proteins for pyocin assembly (Toyofuku et al. 2014).

#### Cultivation under osmotic stress

Osmotic stress is another condition related to high vesiculation, caused by changes in pH and the addition of salts to equilibrate it (Baumgarten et al. 2012; Lee et al. 2018). Exposure of *L. monocytogenes* to NaCl, 0.5 M, increases the release of vesicles, obtaining spherical nanostructures without appreciable differences in shape and size compared to the control (Lee et al. 2018). Significant increases of OMVs were also reported in *P. putida* cultures upon 2 M of NaCl (Baumgarten et al. 2012).

The increase in the concentration of NaCl in the extracellular environment of *L. monocytogenes* activated the responses to stress σ^B^ and SOS, being found in the MVs proteins related to both stress systems, such as osmolyte transporters or RecA (Lee et al. 2018). Likewise, the stress condition increased the concentration of virulence factors and proteins related to osmotic stress, such as GbuA (Lee et al. 2018). Meanwhile, vesicles from *P. putida* showed a significant increase in the degree of saturation of fatty acids with respect to the parental cell, coupled with an increase in the hydrophobicity of the cell surface and in the capacity of the bacteria to form biofilms (Baumgarten et al. 2012). Therefore, the increase in vesiculation in *L. monocytogenes* and *P. putida* could be related to alterations in membrane fluidity, protein folding, and DNA changes triggered by the presence of salts (Mozaheb and Mingeot-Leclercq 2020). Based on the protein composition of the MVs of *L. monocytogenes*, the secretion of vesicles might be a survival mechanism through which the bacterium adapts to natural environments such as the small intestine and duodenum, in which the osmotic pressure varies (Lee et al. 2018). Whereas 1% and 2% of NaCl in cultures of *S. aureus* reduce MV secretion compared to the control (culture without NaCl) due to a thickening of the cell wall that prevents the exit of the MVs (Wang et al. 2021a, b).

#### Cultivation under nutrient depletion

Nutrient depletion has been evaluated as an inducer of vesiculation in *N. meningitidis* for the formulation of vaccines against Meningococcus Group B (Gerritzen et al. 2019a; van de Waterbeemd et al. 2013b). For instance, the cysteine limitation increases vesiculation 12-fold compared with the control (van de Waterbeemd et al. 2013b). Likewise, the culture of *N. meningitidis* under reduced concentrations of sulfate increases the number of vesicles two-fold in relation to a condition of cysteine depletion (Gerritzen et al. 2019a).

The morphology of the vesicles secreted by *N. meningitidis* due to nutrient depletion does not present significant variations with respect to the controls (Gerritzen et al. 2019a; van de Waterbeemd et al. 2013b). Additionally, the OMVs generated from the depletion of cysteine have a similar composition to OMVs released by the bacteria during infection, making it possible to use them in the formulation of vaccines against Meningococcus Group B (van de Waterbeemd et al. 2013b). However, the composition of OMVs derived from sulfate depletion presents an enrichment of phospholipids and low concentrations of LPS, which affects their application in vaccine production due to the reduction of PAMPs (Gerritzen et al. 2019a). Transcriptomic analysis of *N. meningitidis* cultured under cysteine-limiting conditions suggests that amino acid depletion alters iron-sulfur protein biogenesis, leading to increased intracellular iron and oxidative stress (van de Waterbeemd et al. 2013b). While sulfate depletion triggers an increase in phospholipid biosynthesis, the accumulation of which may be associated with BEV biogenesis (Gerritzen et al. 2019a). For *S. aureus*, cultured in the presence of the iron chelator 2,2-dipyridyl leads to the production of MVs three-fold higher than the control condition (Wang et al. 2021a, b). The release of BEVs is probably related to acquiring the nutrient (Wang et al. 2021a, b). Table 3 presents stress conditions that increase the production of BEVs, which might be effective for the bioprocesses design.

#### Cultivation with chemical compounds

The contact of bacterial cells with chemical compounds, such as solvents, detergents, or chelators, alters the composition and integrity of the membranes (Mozaheb and Mingeot-Leclercq 2020). As a stress-defense mechanism, bacteria responses such as *cis/trans* isomerization of fatty acyl residue in membrane phospholipids, ESRS activation, and vesicle release (Eberlein et al. 2018; Mozaheb and Mingeot-Leclercq 2020). Compounds such as Ethylenediaminetetraacetic acid (EDTA), which sequester calcium and magnesium ions, destabilize the OM, altering its interaction with PG and IM, and triggering the release of BEVs (Balhuizen et al. 2021a; Baumgarten et al. 2012; van de Waterbeemd et al. 2012). EDTA-induced vesicles tend to be less stable than those released spontaneously (Balhuizen et al. 2021a). Ionic and non-ionic detergents such as SDS (Sodium Dodecyl Sulfate) and deoxycholic acid, respectively, provoke increased vesiculation and detoxified OMVs by removing LPS, but with the disadvantage of vesicle aggregation (Gnopo et al. 2017; van de Waterbeemd et al. 2010, 2012). Currently, the generation of genetically modified strains with LPS of attenuated toxicity seeks to replace the production of BEVs mediated by detergents (Gerritzen et al. 2019b; van de Waterbeemd et al. 2013a).

The 1-octanol, ethanol, D-cycloserine, lysine, and sucrose fatty acid esters also increase the production of BEVs, triggering alterations in the PG (Table 3) (Abe et al. 2021; Baumgarten et al. 2012; Wang et al. 2021a, b; Yokoyama et al. 2021). D-cycloserine, analogous to D-alanine, acts as an inhibitor of PG synthesis, altering the integrity of the envelope of *P. aeruginosa* (MacDonald and Kuehn 2013). Lysine reduces the expression of HM1357, which is responsible for controlling the transcription of genes related to PG synthesis in *Shewanella vesiculosa* (Yokoyama et al. 2021). The sucrose fatty acid esters, a surfactant with antimicrobial activity, activate autolysins already expressed in the cell wall of *B. subtilis*, triggering PG degradation and cell death (Abe et al. 2021). The sucrose fatty acid esters cause the greatest increases in vesiculation (Abe et al. 2021). The composition of BEVs seems to be closely linked to the stress response mechanisms activated by the cell in the presence of the above mentioned compounds, where lysine sensor proteins (HM1275) and autolysin were found as part of their response protein profile (Abe et al. 2021; Yokoyama et al. 2021). For instance, the presence of BEVs maintains the viability of *B. subtilis* around 90% in presence of sucrose fatty acid esters up to 40 μg/mL, and the load of HM1275 in BEVs of *S. vesiculosa* was related to an increase in the ability of the bacteria to form biofilms (Abe et al. 2021; Yokoyama et al. 2021). While, with the use of EDTA, an increase in the degree of lipid saturation was reported (Baumgarten et al. 2012).

#### Cultivation with antibiotics

Antibiotics such as gentamicin, ciprofloxacin, chloramphenicol, polymyxin B, and ampicillin, as well as antimicrobial peptides such as cathelicidins or Host Defense Peptides (HDPs) are vesiculation inducers (Balhuizen et al. 2021b; Bos et al. 2021; Fulsundar et al. 2014; Godlewska et al. 2019; Kim et al. 2020b). Antibiotic-mediated vesicle generation is related to the mechanism of action of the compounds on cells. Ciprofloxacin is an inhibitor of DNA replication that causes activation of the SOS response related to the explosive cell lysis and is correlated with the increase of BEVs in *E. coli* (Bos et al. 2021; Turnbull et al. 2016). Ampicillin, an antibiotic of the β-lactam family applied to *S. aureus* cultures, leads to the most significant increases in vesiculation, 22-fold compared to the control (Kim et al. 2020b). The polymyxin B used in *C. jejuni* (Godlewska et al. 2019) and HDPs in *B. bronchiseptica* cultures also moderately increase vesicle production (Balhuizen et al. 2021b). While gentamicin, a polycationic antibiotic that, in addition to inhibiting protein synthesis, interacts with OM, causing alterations in LPS due to the displacement of Ca^2+^ and Mg^2+^, being responsible for the increase in vesiculation sites of *A. baylyi* (Fulsundar et al. 2014; Mozaheb and Mingeot-Leclercq 2020). Morphology and properties of the BEVs of *B. bronchiseptica* in the presence of cathelicidin derived in spherical and tubular vesicles with average sizes between 20 and 40 nm reduce its stability compared to the spontaneous OMVs (Balhuizen et al. 2021b).

The composition of BEVs induced by antibiotics, in most cases, presents an increase in protein concentration, such as enzymes are related to the degradation of the applied antibiotic (Fulsundar et al. 2014; Godlewska et al. 2019; Kim et al. 2020b). The addition of ampicillin to *E. coli* cultures significantly increases the amount of Pal lipoprotein, which, together with LPS, increases the toxicity of BEVs (Michel et al. 2020). LPS in BEVs from *A. baylyi* increases in response to gentamicin, as well as DNA concentration (Fulsundar et al. 2014). The increased accumulation of other lipids, such as phosphatidylglycerol in BEVs from *B. bronchiseptica*, also has been detected (Balhuizen et al. 2021b), changing its interaction with macrophages, reducing the expression of cytokines, and a loss of virulence (Balhuizen et al. 2021b).

#### Considerations for the production of BEVs

One environmental aspect important in the production of BEVs is the culture medium, which can be key in the abundant vesiculation that might determine their composition (McCaig et al. 2016; Yokoyama et al. 2021). The culture of *Haemophilus paresuis* in Brain Heart Infusion increases vesiculation compared to Casman’s broth base and Soy Trypticase media (McCaig et al. 2016). Likewise, the culture of *S. vesiculosa* in Lysogenic Broth (LB) increases the release of BEVs 39-fold compared to Bacto Marine Broth and eight-fold compared to M79 (modified DSMZ medium). The culture of *H. paresuis* in LB was also accompanied by an increase in the production of protein P49, which is abundant in BEVs (Yokoyama et al. 2021). Although the function of this protein is not known, its presence in the vesicles was associated with the non-canonical protein secretion system T2SS and particularly with the GspD2 protein, a possible OM channel responsible for the loading of molecules in the BEVs. The P49 protein is also a candidate to be fused with proteins to be transported and loaded in *S. vesiculosa* vesicles (Chen et al. 2020b). Unlike *B. pertussis* and *B. bronchiseptica* cultured in Verwey, Stainer-Scholte, and Thalen-IJssel media, influence the protein composition of OMVs, finding the receptor FauA (siderophore) in vesicles produced in Verwey, and the Zn receptor, ZnuD, in vesicles derived from Stainer-Scholte, and Thalen-IJssel media (De Jonge et al. 2021), attributed to the low availability of iron in those media (De Jonge et al. 2021).

The mode of operation of the bioprocess and the incubation time also increases the production of BEVs from some bacteria (Gerritzen et al. 2019b; Richter et al. 2021b). The production of vesicles by *N. meningiditis* in stirred tank reactors operated in continuous mode increases the production of BEVs by nine-fold compared to batch culture (Gerritzen et al. 2019b). While the *E. coli* BL21 DE3 culture for seven days leads to a higher concentration of BEVs compared to the culture for two days (Richter et al. 2021b). In the latter case, it is assumed that extending the culture time leads the bacteria to a starvation state resulting in a high release of BEVs (Richter et al. 2021b).

#### Strategies for the formation and recovery of artificial BEVs

Although various methods for abundant vesicle formation during bacterial culture have been tried, large-scale production of BEVs remains challenging. Various strategies have been implemented to generate artificial vesicles to solve this drawback. These strategies involve the use of chemical or physical processes that trigger the death or lysis of bacteria, releasing essential components, like lipid bilayers, proteins, and nucleic acids, for the reconstitution of BEVs (Fig. 4C and 4G) (Hahm et al. 2021; Park et al. 2021).

#### Artificials BEVs production by sonication

Sonication is the most used method in the production of artificial vesicles (Fig. 4D). (Hozbor et al. 1999; Park et al. 2021; Mougenot et al. 2022). Once the cells are isolated, they are resuspended in a buffer (pH between 8 and 8.5) and pretreated with EDTA and lysozyme (Hozbor et al. 1999; Park et al. 2021), weakens the cell envelope and degrading PG, respectively, generating spheroplasts (Li et al. 2021; Park et al. 2021). Protocols include the IM remotion by detergents such as Sarkosyl and cytosolic components by increasing the pH. Then, OMs are purified and sonicated at mild intensities (Fig. S3A) (Park et al. 2021). Sonication increased the number of BEVs compared to spontaneous release about 40-fold in *E. coli* (Park et al. 2021). In general, sonication produces spherical BEVs of around 50—150 nm and 150 and 250 nm (Hozbor et al. 1999; Park et al. 2021), with decreasing compounds such as LPS (Park et al. 2021). The protein profiles differ with respect to spontaneous OMVs, highlighting a higher accumulation of OMPs, the reduction of cytosolic proteins, and a lower capacity to protect cargo molecules (McCaig et al. 2016).

#### Artificial BEV production by extrusion

Extrusion is another methodology to produce artificial BEVs, using strategies such as the generation of protoplasts (Fig. S3B) (Harisa et al. 2020; Kim et al. 2015b). Extrusion involves the passage of protoplasts through a series of polycarbonate membranes of different sizes (10, 5, and 1 µm), obtaining Protoplast-derived nanovesicles (PDNVs) (Kim et al. 2015b). Then, the PDNVs are purified by density gradient ultracentrifugation (Kim et al. 2015b). The PDNVs have represented 200-fold higher than the spontaneous BEVs in *E. coli*. PDNVs present spherical morphologies with average diameters of 114 ± 10 nm, harboring cytoplasmic proteins and lacking OM components, such as LPS or OmpA (Kim et al. 2015b).

The production of vesicles from ghost bacteria has an opposite approach to that of protoplasm (Fig. S3B). In this strategy, the first step consists of generating cell envelopes devoid of cytoplasmic content using the lysis by the E gene (bacteriophage PhiX174) and/or the sponge-like protocol (Harisa et al. 2020; Youssof et al. 2019). The lysis method involves the genetic modification of the bacteria with the E gene, encoding a membrane protein that oligomerizes in a transmembrane tunnel allowing the cytoplasmic content release, and leaving the cell envelope intact (Harisa et al. 2020; Langemann et al. 2010). The sponge-like protocol method uses agents such as sodium hydroxide, calcium carbonate, SDS, H_2_O_2_, and ethanol, which degrade genetic material and other cellular components and promote their release through an altered cell envelope (Youssof et al. 2019). Once the ghost cells are obtained, they are extruded through membranes with pore sizes of 100 nm, obtaining vesicles called bacteriosomes (Harisa et al. 2020).

#### Artificials BEVs production by high pressures

The production of BEVs at high pressures (Fig. S3C), is a method analogous to the extrusion process. In the pressure method, self-assembling vesicles, or bacterial biomimetic vesicles (BBVs) are formed after passing the membranes through a homogenizer with small holes that deform the cell envelope in the form of buds (Hua et al. 2021; Li et al. 2021). Through this method, the number of vesicles produced by *K. pneumoniae* and *E. coli* has increased to 88 and 98-fold, respectively, compared with spontaneously released OMVs (Hua et al. 2021; Li et al. 2021). The BBVs of *E. coli* were also loaded with the fusion protein ClyA-IL10, increasing the amount of ClyA-IL10, 31-fold higher compared to IL10-OMVs (Hua et al. 2021). In addition to increasing vesicle production, this method improves the incorporation of cargo molecules.

The morphology of the BBVs, obtained at a pressure of 1200 bar, is mainly rounded with average sizes between 180 and 210 nm, and stable for at least five weeks at room temperature, 4 °C, and − 20 °C (Hua et al. 2021; Li et al. 2021). At lower pressures (200 to 800 bar), the vesicles present incomplete structures, ruling out these operating conditions (Hua et al. 2021). The protein composition of BBVs shows a significant increase in IM, OM, and periplasmic proteins and a reduction in cytoplasmic components, including nucleic acids (Hua et al. 2021; Li et al. 2021). Both *K. pneumonia* and *E. coli* BBVs, conceived for immunogenic treatments (production of vaccines and anticancer agents, respectively), managed to activate the immune response of the host organisms where they were evaluated, providing a new alternative to produce vaccines and immunotherapy for cancer (Hua et al. 2021; Li et al. 2021).

#### Quantification of BEVs

Quantification is crucial for evaluating the efficiency of BEV production strategies (Klimentová and Stulík 2015). Various analytical methods can be used to quantify BEVs (Tables 2 and 3), which can be divided into direct and indirect (Bitto et al. 2021; Klimentová and Stulík 2015). Indirect methods, such as the determination of total protein and lipids, are the most common (Tables 2 and 3). These have the advantages of easy implementation, not requiring sophisticated equipment, and generally being a routine laboratory analysis. The most used protein assays are Bradford and bicinchoninic acid (BCA) (Bitto et al. 2021; Klimentová and Stulík 2015). Meanwhile, for the quantification of BEVs through lipids, the FM4-64 assay is widely reported (Klimentová and Stulík 2015). However, it is not always possible to correlate the protein and lipid content with the BEV quantity since the composition and size of the vesicles may vary depending on their production conditions, bacterial strain, and isolation method, among others (Hirayama and Nakao 2020; Bitto et al. 2021; Wei et al. 2022). Dry weight has been reported as a relative measure of vesiculation (Deatherage et al. 2009; Klimentová and Stulík 2015; McMahon et al. 2012). However, this technique can measure cellular material that does not correspond to vesicles (McMahon et al. 2012).

Direct methods, such as nanoparticle tracking Analysis (NTA), flow cytometry, transmission electron microscopy (TEM), or tunable resistive pulse sensing, are also used for vesicle quantification (Bitto et al. 2021; Goreham et al. 2019), among others. These methods are interesting because they allow the direct counting of particles, ensuring that changes in BEV composition would not affect possible comparisons between production processes (Bitto et al. 2021). However, the disadvantages lie in the need for expensive equipment and specialized staff (Goreham et al. 2019). It is necessary to continue the search for accessible methods that allow direct quantification of BEVs, limiting the biases introduced by indirect methods.

## Concluding remarks

The biological relevance of BEVs has been recognized, moreover its use and biotechnological application continue to be in development. Here, we focus on strategies that increase vesicle production, inspired by BEV biogenesis mechanisms and functions, with significant increases in vesiculation. Having to mention that in several studies, different families of vesicles are observed; each of them might have different protein patterns and number of molecules inside, the lipid composition changes, and the nature of the vesicles varies according to the culture time, production time, and in response to the changes in environmental conditions suffered during production. Furthermore, we described different insults that cause hypervesiculating phenotypes. However, until now, mutants that do not vesiculate have been described, pointing out that this mechanism is constant in cells and could be manipulated and orchestrated as a cellular response.

A constant debate, with a view to the biotechnological use, is the low amounts of OMVs that are obtained from cultured bacteria at least two or three orders of magnitude with respect to the biomass. This is combined with the variation in the formation of vesicles and technical difficulties in the biophysical determination of their properties. Even more, during their purification, vesicles might aggregate or coalesce and are influenced by known purification methods. Some results published works may contain artifacts, or the physicochemical analysis only describes a part of a variety of BEVs that could be obtained in production processes.

Biotechnologically, it is still important to know the biological background of production to design OMVs with known and designed characteristics. In the future, the knowledge that might be generated about BEV production, the importance of environmental conditions on it, the kinetic analysis, as well as the use of in-depth lipidomic, proteomic, and microscopy techniques will allow improvement in the knowledge and description of the BEVs from relevant strains to clinical evaluations. Importantly, the sum of knowledge on their properties, and the application needs, could lead to controlled bioprocesses producing BEVs with known morphologies, up to the design of composition, shape, and size that will ensure their quality, safe, and effectiveness.

### Supplementary Information

Below is the link to the electronic supplementary material.

Supplementary file1 (DOCX 1311 KB)

## Data Availability

No datasets were generated or analyzed during the current study.
