# Supplementary material for: Bacterial extracellular vesicles: biotechnological perspective for enhanced productivity
Source: World J Microbiol Biotechnol. 2024 Apr 20;40(6):174. doi: 10.1007/s11274-024-03963-7 (PMC11032300; doi:10.1007/s11274-024-03963-7)
Supplement: Supplementary file 1 — Supplementary file1 (DOCX 1311 KB) [file 11274_2024_3963_MOESM1_ESM.docx]

**Supplementary material**


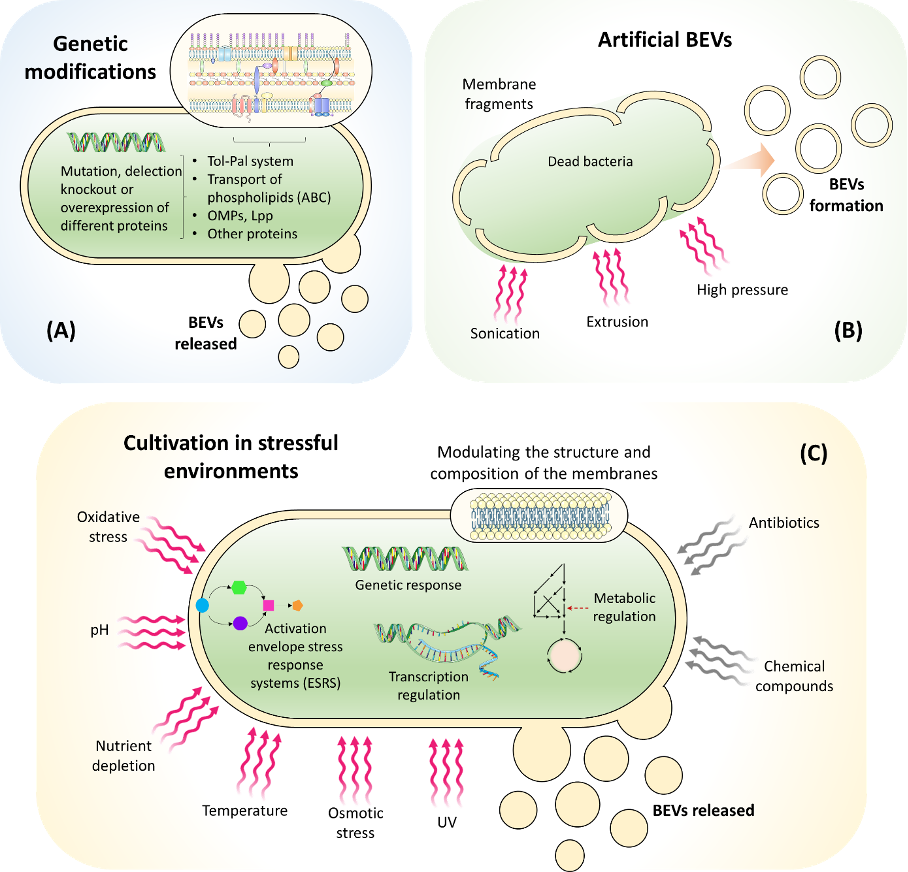


Figure S1. Strategies to increase the productivity of BEVs (A) Genetic modifications of genes associated with proteins or processes of the cell envelope. (B) Formation and recovery of artificial BEVs. (C) Cultivation in stressful environments.


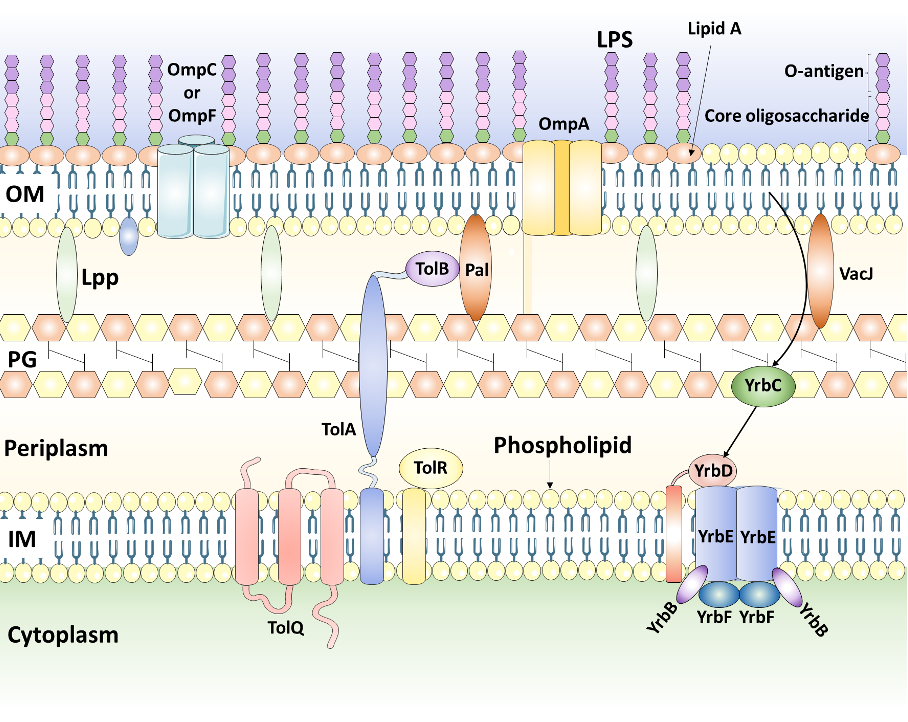


**Figure S2.** Gram-negative cell wall structure and protein targets used in hypervesiculation bacteria strains development. Gram-negative cell wall is composed of inner and outer membranes (IM and OM), and a thin layer of peptidoglycan (PG) embedded in the periplasm. The OM has outer membrane proteins (OMPs). OM and PG are cross-linked by lipoproteins (Lpp). Outer leaf in outer membrane is composed by lipopolysaccharides (LPS). Among target proteins to obtain strains with hypervesiculation can be found the Tol-Pal system, specific lipoproteins, chaperones, and the ABC transporter VacJ/Yrb. Created with Servier Medical Art resources.


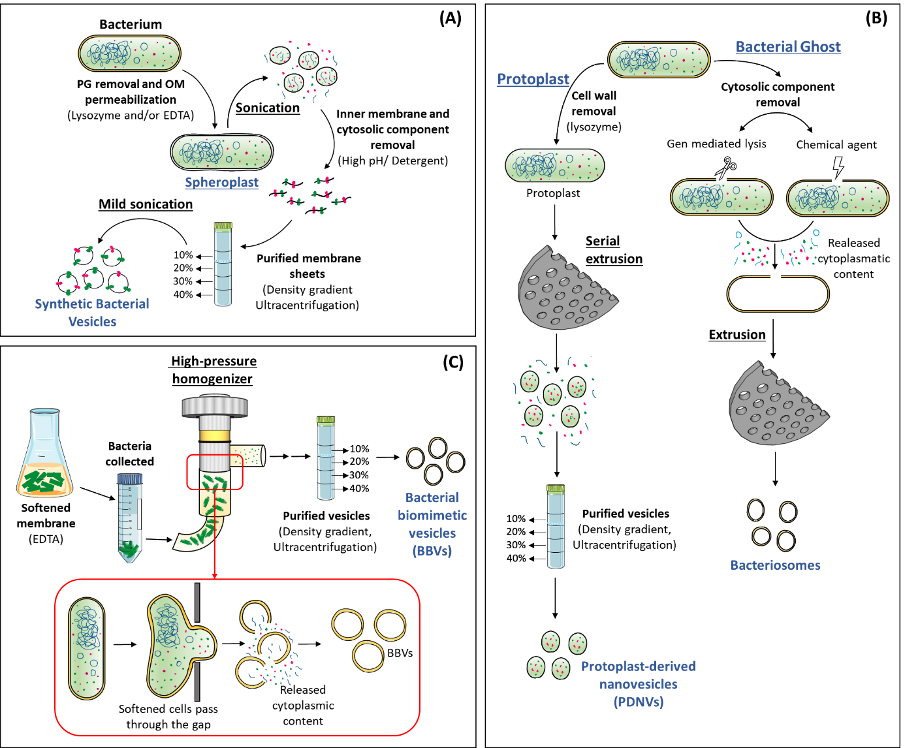


**Figure S3.** Production and recovery methodologies of synthetic BEVs. **(A)** Cells sonication to obtain synthetic bacterial vesicles. **(B)** Extrusion of protoplasts and ghost bacteria, to obtain protoplast-derived nanovesicles (PDNVs) and bacteriosomes, respectively. **(C)** High pressure to obtain biomimetic bacterial vesicles (BBVs). The figure was created using Servier Medical Art resources.
